# Root-knot nematode infection enhances the performance of a specialist root herbivore via plant-mediated interactions

**DOI:** 10.1093/plphys/kiaf109

**Published:** 2025-03-20

**Authors:** Axel J Touw, Nhu Tran, Andreas Schedl, Jessil A Pajar, Cong Van Doan, Henriette Uthe, Nicole M van Dam

**Affiliations:** Plant Biotic Interactions, Leibniz Institute of Vegetable and Ornamental Crops (IGZ), Theodor-Echtermeyer-Weg 1, D-14979 Großbeeren, Germany; Institute of Biodiversity, Friedrich Schiller University Jena, Dornburger Str. 159, D-07743 Jena, Germany; Molecular Interaction Ecology, German Centre for Integrative Biodiversity Research (iDiv) Halle-Jena-Leipzig, Puschstraße 4, D-04103 Leipzig, Germany; Molecular Interaction Ecology, German Centre for Integrative Biodiversity Research (iDiv) Halle-Jena-Leipzig, Puschstraße 4, D-04103 Leipzig, Germany; Institute for Plant Sciences, University of Cologne, Cluster of Excellence on Plant Science (CEPLAS), Zülpicher Str. 47b, D-50674 Cologne, Germany; Molecular Interaction Ecology, German Centre for Integrative Biodiversity Research (iDiv) Halle-Jena-Leipzig, Puschstraße 4, D-04103 Leipzig, Germany; Biogenic Char Applications, Deutsches Biomasseforschungszentrum gemeinnützige GmbH (DBFZ), Torgauer Str. 116, D-04347 Leipzig, Germany; Plant Biotic Interactions, Leibniz Institute of Vegetable and Ornamental Crops (IGZ), Theodor-Echtermeyer-Weg 1, D-14979 Großbeeren, Germany; Institute of Biodiversity, Friedrich Schiller University Jena, Dornburger Str. 159, D-07743 Jena, Germany; Molecular Interaction Ecology, German Centre for Integrative Biodiversity Research (iDiv) Halle-Jena-Leipzig, Puschstraße 4, D-04103 Leipzig, Germany; Chemical Communication in Ecological Systems (CCES), International Max Planck Research School (IMPRS), Hans-Knöll-Straße 8, D-07745 Jena, Germany; Plant Biotic Interactions, Leibniz Institute of Vegetable and Ornamental Crops (IGZ), Theodor-Echtermeyer-Weg 1, D-14979 Großbeeren, Germany; Plant Physiology Unit, Department of Life Sciences and Systems Biology, University of Turin, Via Accademia Albertina 13, 10123 Torino, Italy; Molecular Interaction Ecology, German Centre for Integrative Biodiversity Research (iDiv) Halle-Jena-Leipzig, Puschstraße 4, D-04103 Leipzig, Germany; MetaCom Metabolomics Facility, Leibniz Institute of Plant Biochemistry (IPB), Weinberg 3, D-06120 Halle (Saale), Germany; Plant Biotic Interactions, Leibniz Institute of Vegetable and Ornamental Crops (IGZ), Theodor-Echtermeyer-Weg 1, D-14979 Großbeeren, Germany; Institute of Biodiversity, Friedrich Schiller University Jena, Dornburger Str. 159, D-07743 Jena, Germany; Molecular Interaction Ecology, German Centre for Integrative Biodiversity Research (iDiv) Halle-Jena-Leipzig, Puschstraße 4, D-04103 Leipzig, Germany

## Abstract

Herbivores sharing host plants are often temporally and spatially separated, limiting direct interactions between them. Nevertheless, as observed in numerous aboveground study systems, they can reciprocally influence each other via systemically induced plant responses. In contrast, examples of such plant-mediated interactions between belowground herbivores are scarce; however, we postulated that they similarly occur, given the large diversity of root-interacting soil organisms. To test this hypothesis, we analyzed the performance of cabbage root fly (*Delia radicum*) larvae feeding on the main roots of field mustard (*Brassica rapa*) plants whose fine roots were infected by the root-knot nematode (*Meloidogyne incognita*). Simultaneously, we studied the effects of *M. incognita* on *D. radicum*-induced defense responses and the accumulation of primary metabolites in the main root. We observed that almost 1.5 times as many *D. radicum* adults emerged from nematode-infected plants, indicating a facilitation effect of *M. incognita* infection. Although we observed increases in the accumulation of proteins and 2 essential amino acids, the strongest effect of nematode infection was visible in the defense response to *D. radicum*. We observed a 1.5 times higher accumulation of the defense-related phytohormone JA-Ile in response to *D. radicum* on nematode-infected plants, coinciding with a 75% increase in indole glucosinolate concentrations. Contrastingly, concentrations of aliphatic glucosinolates, secondary metabolites negatively affecting *D. radicum*, were 10% to 25% lower in nematode-infected plants. We hypothesize that the attenuated aliphatic glucosinolate concentrations result from antagonistic interactions between biosynthetic pathways of both glucosinolate classes, which was reflected in the expression of key biosynthesis genes. Our results provide explicit evidence of plant-mediated interactions between belowground organisms, likely via systemically induced responses in roots.

## Teaser

*Delia radicum* larvae feeding on nematode-infected *Brassica rapa* roots have higher performance due to reduced chemical defenses and increased primary metabolite and protein content in infested plants.

## Introduction

Plants form the basis of most natural ecosystems and interact with a highly diverse community of organisms. Belowground, soil-borne organisms, such as insect herbivores, bacteria, fungi, oomycetes, and plant-parasitic nematodes, rely on roots as an essential source of carbohydrates, amino acids (AAs), proteins, and fatty acids. Based on root function and ontogeny, concentrations of these primary metabolites differ among root classes. In dicots with a taproot system, primary metabolites such as carbohydrates are usually present at the highest concentrations in the main root (Hennion et al. 2019), which consequently is the most attractive root part for herbivores and pathogens. Nevertheless, root herbivores do not necessarily target the main root exclusively (reviewed in Tsunoda and van Dam 2017). Belowground organisms are differently affected by soil conditions (Brown and Gange 1990; Eilers et al. 2012; Becerra et al. 2014; Liu et al. 2022) as well as physical (Johnson et al. 2010; Holbein et al. 2019; Kawasaki et al. 2021) and chemical root traits (Robert et al. 2012; Teixeira et al. 2016; Tsunoda et al. 2017), and therefore interact with distinct parts of the root system. As a result, members of the root-associated organismal community are often spatially separated, with limited direct interactions. However, because root herbivory induces both local and systemic responses (Tsunoda et al. 2018; Mbaluto et al. 2021), organisms feeding on distinct root parts may affect each other's performance via changes in the shared host plant.

Plant-mediated interactions occur when 1 organism induces phenotypic changes in the plant that affect the performance of a subsequent interactor (Paul et al. 2000; Heil and Ton 2008; Stam et al. 2014). Over the last decades, numerous studies have addressed such interactions, whereby systemically induced changes in the primary metabolome (Hol et al. 2013) or secondary metabolome (Hol et al. 2004; Soler et al. 2007, 2012; Kroes et al. 2015; Karssemeijer et al. 2020; Mbaluto et al. 2021) were considered the underlying cause. The outcome of plant-mediated interactions may range from inhibition to facilitation, depending on the nature of the inducing organism, plant genotype, and the responding organism (Lu et al. 2016). As illustrated by the examples above, most studies addressing plant-mediated interactions have focused on aboveground organisms or interactions between root- and shoot-feeding herbivores. In contrast, the number of studies analyzing belowground plant-mediated interactions is limited. However, because soils host a high diversity of organisms (Bardgett and van der Putten 2014), and plant roots are important foundations of belowground communities (Wardle 2006), belowground plant-mediated interactions are likely just as common.

In this study, we assessed belowground, plant-mediated interactions between the root-knot nematode (*Meloidogyne incognita*; hereafter RKN) and the cabbage root fly (*Delia radicum*), sharing field mustard (*Brassica rapa*) as a host plant. We selected these herbivores because both are important agricultural pests and because they interact with distinct parts of the root system. The larvae of the cabbage root fly, a specialist in brassicaceous plants, feed mostly on the main root (Tsunoda et al. 2017). In contrast, *M. incognita* mainly infects fine and lateral roots. The ubiquitous nematode *M. incognita* is a generalist that infects almost every family of vascular plants, including Brassicaceae (Jones et al. 2013; Hol et al. 2016; Eisenback 2020). It is, therefore, likely that both herbivores regularly share host plants. However, because of their preference for distinct parts of the root system, any interactions between nematodes and root fly larvae likely result from systemically induced changes in their shared host.

*M. incognita* may induce multiple phenotypic changes in the plant with the potential to affect *D. radicum* performance. After entering the root at the elongation zone, infective juveniles move toward the root tip, enter the vascular cylinder, and subsequently move upwards to settle in the differentiation zone. There it induces the formation of a giant cell, a structure commonly known as a root-knot (Wyss et al. 1992; Wondafrash et al. 2013; Hiltpold et al. 2015). To successfully establish its feeding site, *M. incognita* suppresses defense responses by excreting effector proteins that target jasmonic acid (JA) (Ji et al. 2013) and salicylic acid (SA) signaling (Wang et al. 2018). The suppression of the JA-regulatory pathway attenuates defense responses targeted at *M. incognita* in roots, whereas several studies have recorded alterations of the phytohormonal response in shoot tissues as well (Van Dam et al. 2018; Mbaluto et al. 2021). Most studies addressing aboveground–belowground interactions between RKNs and chewing or sucking shoot herbivores recorded that RKN-infection either has neutral effects or enhances the performance of insect herbivores (Kaplan et al. 2011; Hol et al. 2013; Mbaluto et al. 2021). It was suggested that this is because RKNs not only suppress plant defense responses locally but also systemically, benefiting shoot-feeding insects that would otherwise be affected by the JA-regulated defense responses they themselves elicit (Wondafrash et al. 2013). Similar plant-mediated interaction processes may govern interactions between RKNs and root-feeding insect herbivores. *D. radicum* feeding mainly elicits the JA signaling pathway (Karssemeijer et al. 2022), whereby increases in JA and JA-Ile levels are followed by an increase in glucosinolate levels, secondary metabolites characteristic for Brassicaceae (Fahey et al. 2001). Even though the larvae of *D. radicum* feed specifically on Brassicaceae, the isothiocyanates resulting from the conversion of GSLs upon root damage still reduce *D. radicum* larval performance (Crespo et al. 2012; Sontowski et al. 2022). In case the suppression of JA signaling by *M. incognita* extends to systemic tissues, *M. incognita* infection may lead to decreased levels of GSLs and other JA-regulated defenses in the main root, which can facilitate *D. radicum*.

A second effect of *M. incognita* infection that can promote *D. radicum* performance is the increased accumulation of primary metabolites in the main root. After arriving at the differentiation zone of the root, *M. incognita* starts secreting effectors that manipulate pathways of hormones involved in root development, mainly auxin. It also produces hormones itself, specifically cytokinins, which induce nutrient mobilization and delay root senescence (reviewed in Gheysen and Mitchum 2019). The resulting modulation of root development helps the formation of the root-knot and converts it into a metabolic sink, diverting the flow of photosynthates and other nutrients toward the nematode (De Meutter et al. 2003). By increasing the flow of primary metabolites toward the root-knot, nematode infection may increase the concentration of soluble sugars and AAs in the entire root system (Hofmann et al. 2010), thereby enhancing the performance of *D. radicum* larvae (Hopkins et al. 1999). To our knowledge, no study so far has explicitly addressed interactions between RKNs and below-ground chewing herbivores. Still, some evidence exists that herbivory in distal parts of the root system may influence the performance of herbivores feeding from the main root. For example, milkweed beetles (*Tetraopes tetraophthalmus*) feeding on the main root of the common milkweed (*Asclepias syriaca*), of which the fine roots are damaged by wireworms (*Hypnoides abbreviates*) have a higher larval mass compared to those feeding from undamaged plants (Erwin et al. 2013). It was suggested, but not measured, that the effect of herbivory on the fine roots caused a reallocation of photosynthates to the main root, thereby increasing the body mass of larvae feeding in the main roots.

Given its potential effects on the accumulation of primary metabolites (Hofmann et al. 2010) and modification of (induced) defenses (Mbaluto et al. 2021), we hypothesized that infection by the root-knot nematode *M. incognita* would facilitate the development of *D. radicum* larvae through plant-mediated interactions. To test this hypothesis, we combined bioassays to assess the effects on *D. radicum* performance with targeted and untargeted chemical analyses to obtain a comprehensive view of the metabolic response to herbivory by *D. radicum* in RKN-infected plants. In addition, we studied the effects of *M. incognita* infection on the early defense responses against *D. radicum* in terms of phytohormone accumulation and the expression of key genes involved in glucosinolate biosynthesis. We found that relatively more *D. radicum* adults emerged from RKN-infected plants, indicating that the conditions were more favorable for larval development. The increased performance of *D. radicum* coincided with an attenuated defense response in RKN-infected plants regarding aliphatic GSL accumulation and an increased accumulation of proteins and multiple essential AAs. Together, the results of this study demonstrate that RKN-infection can facilitate *D. radicum* performance, thereby providing evidence of plant-mediated interactions between belowground organisms, likely through systemic induced responses in the roots.

## Results

### Higher relative emergence rates and adult weight for *D. radicum* larvae developing on RKN-infected plants

**Figure 1.**
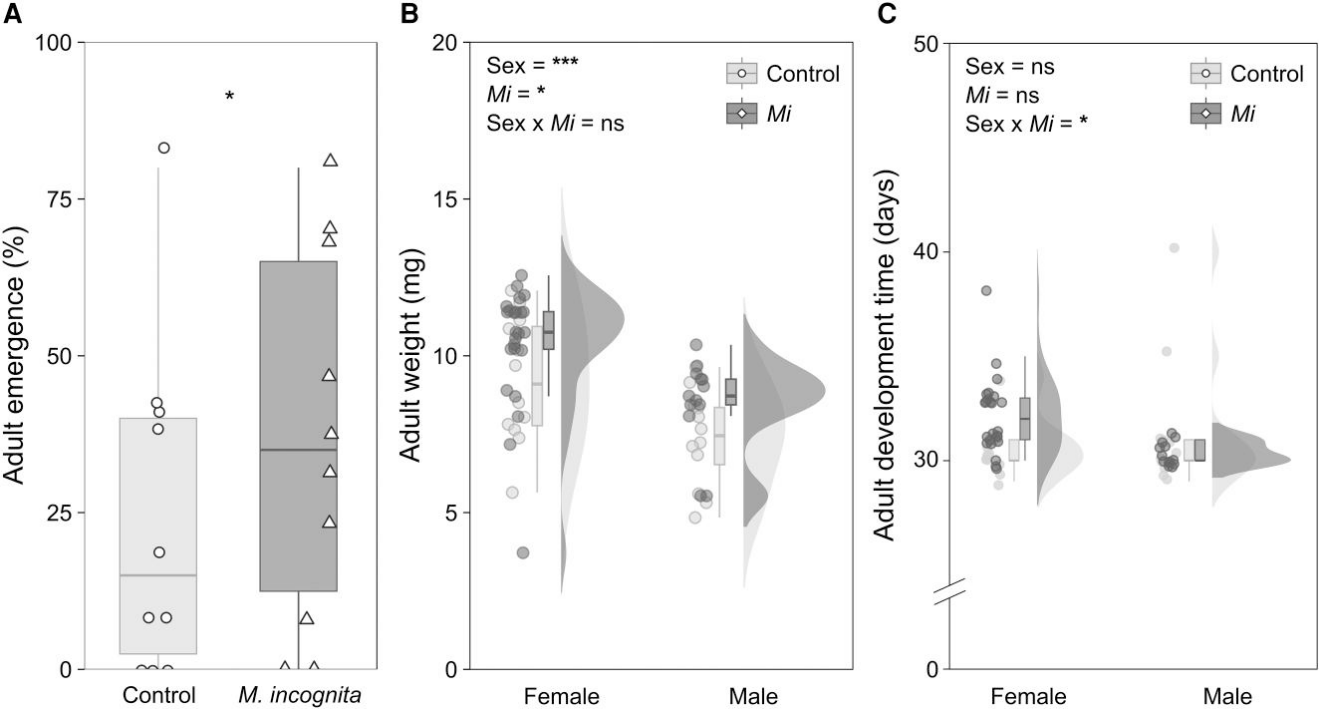
Effects of root-knot nematode infection on cabbage root fly performance. **A)** Percentage of *Delia radicum* adults emerging from eggs (10 eggs per plant) placed on control plants or on plants previously infected by *Meloidogyne incognita* (*Mi*) (*n* = 10). **B)** Body mass (mg) of emerged female and male adults. **C)** Development time (days) of emerged female and male adults. Boxplots represent 25th to 75th percentiles and median, whiskers the 10th and 90th percentiles. Symbols represent individual data points per treatment group. Clouds represent data distribution. Asterisks represent statistical significance (*P < 0.05; ***P < 0.001) in case of adult emergence according to a generalized linear model with Poisson distribution, and for the body mass and development time to a linear model with *Mi*, *Dr* and time point as factors.

To test our hypothesis that any effects of RKN-infection on *D. radicum* performance were the result of plant-mediated interactions, we first confirmed that *M. incognita* infection was indeed restricted to the fine roots by comparing infection rates between fine and main roots (Supplementary Fig. S1A, Supplementary Table S1; *P* < 0.001, F_1_ = 17.197). Next, we addressed the effects of *M. incognita* infection on *D. radicum* performance in a bioassay. Relatively more *D. radicum* adults emerged from RKN-infected plants than from control plants (Fig. 1A, Supplementary Table S2; *P* < 0.05, χ^2^ = 4.6667, df = 1). This higher emergence rate coincided with a higher body mass for both male and female flies (Fig. 1B, *P* = 0.019, F_1_ = 5.8497), whereby females, on average, were heavier than males (Fig. 1B, Supplementary Table S3; *P* < 0.001, F_1_ = 16.1089). The development time from egg to adult was longer on RKN-infected plants, particularly for females (Fig. 1C, interaction sex × *Mi* effects; *P* < 0.05, F_1_ = −2.518), whereby females had an overall longer development time than males (*P* < 0.05, F_1_ = 2.613).

### Nematode infection only minimally affects primary metabolite levels in the main root

**Figure 2.**
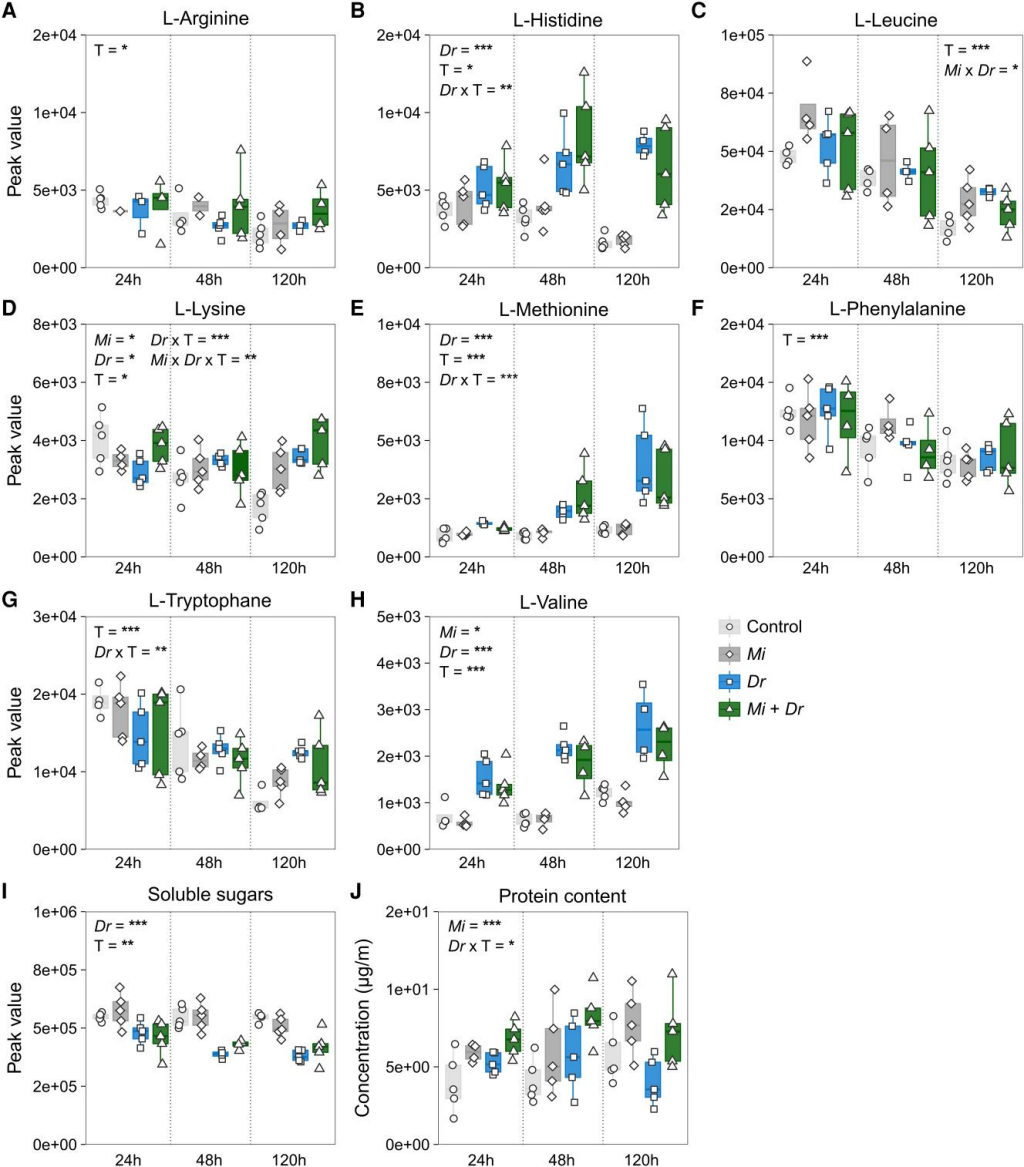
Effects of *Meloidogyne incognita* infection and *Delia radicum* herbivory on the accumulation of primary metabolites in main roots of *Brassica rapa.* Estimates of **A** to **H)** essential amino acids (peak value), **I)** soluble sugars (peak value), and **J)** protein (*µ*mol mg^−1^) present in the main root of control plants (control), plants infected by *Meloidogyne incognita* (*Mi*), plants infested by *Delia radicum* (*Dr*), or plants infected by *M. incognita* and infested by *D. radicum* (*Mi* + *Dr*) measured at time points (T) 24, 48, and 120 h after the start of *D. radicum* herbivory. Boxplots represent 25th to 75th percentiles and median, whiskers the 10th and 90th percentiles. Symbols represent individual replicates per treatment group (*n* = 5). Asterisks depict statistical significance according to 3-way ANOVA: *P < 0.05, **P < 0.01, ***P < 0.001. Only statistically significant effects are depicted in the graph panels.

**Figure 3.**
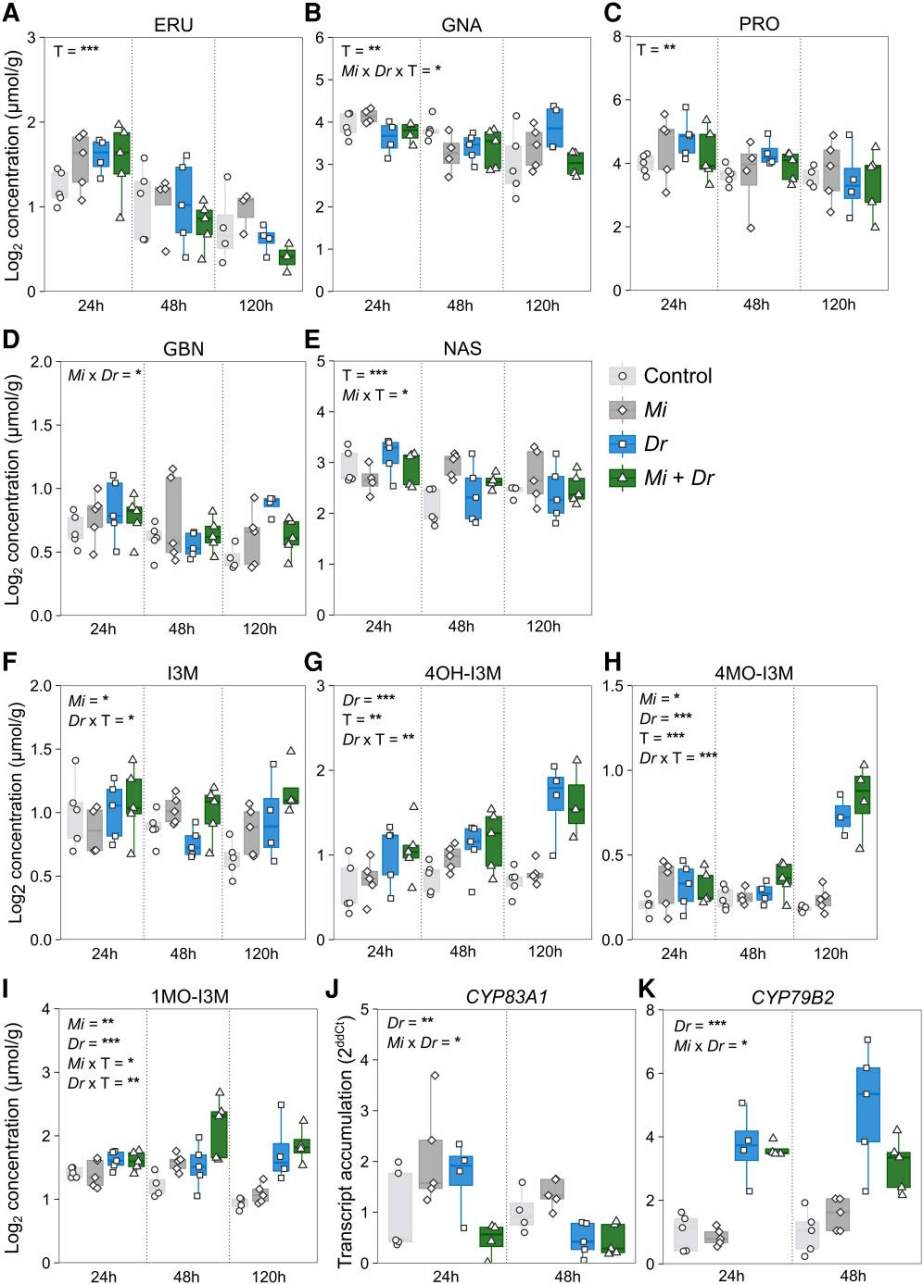
Effects of *Meloidogyne incognita* infection and *Delia radicum* herbivory on glucosinolate accumulation and the expression of glucosinolate biosynthesis genes in main roots of *Brassica rapa.* Main root concentrations (log_2_ *µ*mol g^−1^) of the aliphatic glucosinolates (GSLs) **A)** glucoerucin (ERU), **B)** gluconapin (GNA), **C)** progoitrin (PRO), **D)** glucobrassicanapin (GBN), **E)** the benzenic GSL gluconasturtiin (NAS), and the indole GSLs **F)** indol-3-ylmethyl-GSL (I3M), **G)** 4-hydroxy-indol-3-ylmethyl-GSL (4OH-I3M), **H)** 4-methoxy-indol-3-ylmethyl-GSL (4MO-I3M), and **I)** 1-methoxy-indol-3-ylmethyl-GSL (1MO-I3M). Transcription accumulation (2^ddCt^) of genes involved in biosynthesis of **J)** aliphatic (*CYP83A1*) and **K)** indole (*CYP79B2*) GSLs. GSL accumulation and gene expression were measured in the main root of control plants (control), plants infected by *Meloidogyne incognita* (*Mi*), plants infested by *Delia radicum* (*Dr*), or plants infected by *M. incognita* and infested by *D. radicum* (*Mi* + *Dr*) as measured at time points (T) 24, 48, and 120 h after the start of *D. radicum* herbivory. Boxplots represent 25th to 75th percentiles and median, whiskers the 10th and 90th percentiles. Symbols represent individual replicates per treatment group (*n* = 5). Asterisks depict statistical significance according to 3-way ANOVA: *P < 0.05, **P < 0.01, ***P < 0.001. Only statistically significant effects are depicted in the graph panels.

Comparison of the metabolomes in the main root of different treatments showed that, although herbivory by *D. radicum* was the strongest driver of separation, nematode infection also affected the composition of the main root metabolome (Supplementary Fig. S2). We detected 13 AAs in our extracts (for the total set of AAs, see Supplementary Table S4), 8 of which are considered essential for insect development (Chapman 2012). We focused on these 8 AAs in the main text, whereas the results of the 5 nonessential AAs can be found in Supplementary Fig. S3. Overall, the levels of AAs and sugars in main roots varied over time (Fig. 2A-I, T; *P* < 0.05, Supplementary Table S5), whereas protein levels were more stable (Fig. 2J, *P* > 0.05). Except for arginine and phenylalanine, all AAs, sugar and protein levels responded to at least 1 treatment. We found that herbivory by *D. radicum* alone increased the concentration of the essential AAs histidine (Fig. 2B, *Dr*; *P* < 0.001, F_1_ = 63.904), lysine (Fig. 2D, *P* < 0.05, F_1_ = 6.333), methionine (Fig. 2E, *P* < 0.001, F_1_ = 53.854), tryptophane (Fig. 2G, *P* < 0.01, F_1_ = 6.0928), and valine (Fig. 3H, *P* < 0.001, F_1_ = 125.8492), as well as that of the nonessential AA serine (Supplementary Fig. S3D, *P* < 0.01, F_1_ = 12.0353). Feeding by *D. radicum* larvae decreased asparagine concentrations (Supplementary Fig. S3A, *Dr*; *P* < 0.05, F_1_ = 3.439). RKN-infection alone positively affected concentrations of lysine (Fig. 2D, *Mi*; *P* < 0.05, F_1_ = 4.188) and serine (Supplementary Fig. S3D, *P* < 0.05, F_1_ = 5.4747) and negatively affected valine concentrations (Fig. 2H, *P* < 0.05, F_1_ = 4.449) in main roots. We observed an interaction effect between RKN-infection and *D. radicum* herbivory which resulted in a higher concentration of lysine (Fig. 2D, interaction effect *Mi* × *Dr*; *P* < 0.01, F_1_ = 5.1197) and the nonessential amino acid serine (Supplementary Fig. S3D, *P* < 0.05, F_1_ = 4.3027), and lower concentrations of leucine (Fig. 2C, *P* < 0.05 F_1_ = 5.768), asparagine (Supplementary Fig. S3A, *P* < 0.05, F_1_ = 5.521), and proline (Supplementary Fig. S3D, *P* < 0.01, F_1_ = 9.5082). In addition, accumulation of soluble sugars was negatively affected by *D. radicum* herbivory (Fig. 2I, *Dr*; *P* < 0.001, F_1_ = 74.632), but was not affected by RKN-infection (*Mi*; *P* = 0.5337, F_1_ = 0.5337). Lastly, we observed that RKN-infection increased total protein content (Fig. 2J, *Mi*; *P* < 0.001, F_1_ = 21.849), whereas *D. radicum* herbivory led to reduced protein levels after 120 h (interaction effect *Dr* xT; *P* < 0.05, F_1_ = 3.711), in particular in absence of RKNs.

### *Meloidogyne incognita* infection modulates glucosinolate accumulation in local and distal root tissues during *D. radicum* herbivory

To study the local effects of *M. incognita* on the defense response, we first analyzed GSL concentrations in the fine roots. We found that *M. incognita* infection induced local increases in concentrations of progoitrin (PRO; Supplementary Fig. S4C, Supplementary Table S6; *Mi*; *P* < 0.05, F_1_ = 6.375), gluconasturtiin (NAS; Supplementary Fig. S4D, *Mi*; *P* < 0.001, F_1_ = 32.587), 1-methoxy-indol-3-ylmethyl-GSL (1MO-I3M; Supplementary Fig. S4F, *Mi*; *P* < 0.01, F_1_ = 11.181), and 4-hydroxy-indol-3-ylmethyl-GSL (4OH-I3M; Supplementary Fig. S4G, *Mi*; *P* < 0.05, F_1_ = 6.718). Herbivory by *D. radicum* in the main root increased levels of 1MO-I3M (Supplementary Fig. S4F, *Dr*; *P* < 0.05, F_1_ = 6.858) and 4OH-I3M (Supplementary Fig. S4G, *Dr*; *P* < 0.05, F_1_ = 5.247) in the fine roots. The combined treatment of *M. incognita* and *D. radicum* led to reduced concentrations of glucoerucin (ERU; Supplementary Fig. S4A, *Mi* × *Dr*; *P* < 0.05, F_1_ = 6.265) and gluconapin (GNA; Supplementary Fig. S4B, *Mi* × *Dr*; *P* < 0.05, F_1_ = 4.130), and increased levels of PRO (Supplementary Fig. S4C, *Mi*  *×Dr*; *P* < 0.05, F_1_ = 4.130), 1MO-I3M (Supplementary Fig. S4F, *Mi* × *Dr*; *P* < 0.05, F_1_ = 4.483), and 4-methoxy-indol-3-ylmethyl-GSL (4MO-I3M; Supplementary Fig. S4H, *Mi* × *Dr*; *P* < 0.05, F_1_ = 6.203). Concentrations of indol-3-ylmethyl-GSL (I3M; Supplementary Fig. S4E) in the fine roots were not affected by any of the treatments.

**Figure 4.**
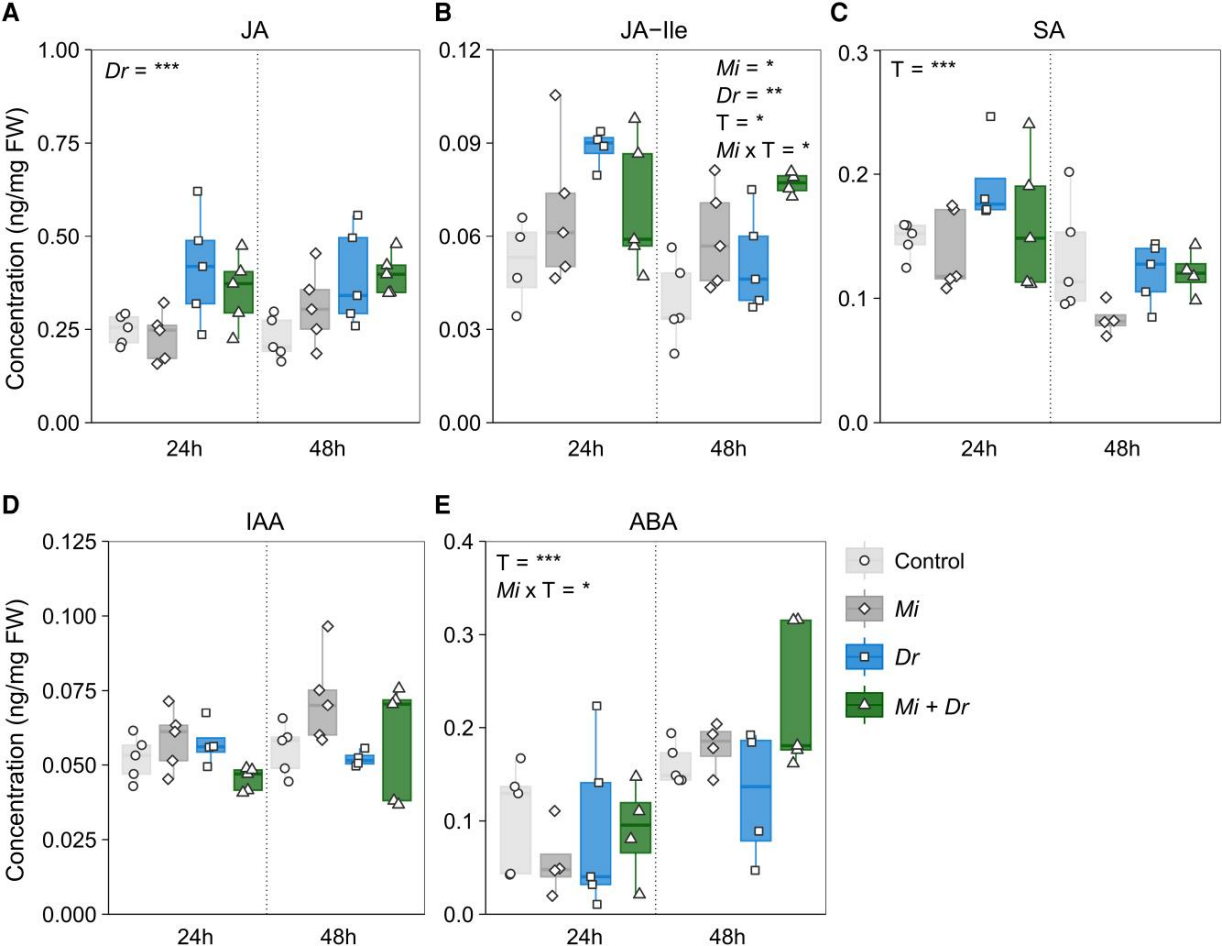
Effects of *Meloidogyne incognita* infection and *Delia radicum* herbivory on phytohormone accumulation in main roots of *Brassica rapa.* Mean concentration (ng mg^−1^) of the jasmonates **A)** jasmonic acid (JA) and **B)** jasmonic acid isoleucine (JA-Ile), **C)** salicylic acid (SA), **D)** indole acetic acid (IAA), and **E)** abscisic acid (ABA) in the main root of control plants (control), plants infected by *Meloidogyne incognita* (*Mi*), plants infested by *Delia radicum* (*Dr*), or plants infected by *M. incognita* and infested by *D. radicum* (*Mi* + *Dr*) as measured at time points (T) 24 and 48 h after the start of *D. radicum* herbivory. Boxplots represent 25th to 75th percentiles and median, whiskers the 10th and 90th percentiles. Symbols represent individual replicates per treatment group (*n* = 5). Asterisks depict statistical significance according to 3-way ANOVA: *P < 0.05, **P < 0.01, ***P < 0.001. Only statistically significant effects are depicted in the graph panels.

Next, we analyzed the accumulation of GSLs and the expression of genes involved in their biosynthesis in the main root. We paid particular attention to differences in aliphatic and benzenic GSLs concentrations since their breakdown products are known to negatively affect the performance of above—and below-ground chewing insects, including *D. radicum* (Sontowski et al. 2022). Concentrations of several aliphatic (Fig. 3, Supplementary Table S5; ERU (Fig. 3A), GNA, (Fig. 3B), PRO (Fig. 3C)), benzenic GSLs (Fig. 3E; NAS) and indole (Fig. 3; 4OH-I3M, (Fig. 3G) and 4MO-I3M (Fig. 3H)) varied over time (T; *P* < 0.05). Levels of aliphatic and benzenic GSL thereby tended to decrease with time, whereas those of indole GSL remained stable or increased (Fig. 3). We observed that neither RKN-infection nor root herbivory individually affected concentrations of aliphatic GSLs in the main root. However, the combined treatment of RKN-infection and *D. radicum* herbivory led to lower concentrations of GNA (Fig. 3B, interaction effect *Mi* × *Dr* × *T*; *P* < 0.05, F_1_ = 4.984) and glucobrassicanapin (GBN, Fig. 3D, interaction effect *Mi* × *Dr*; *P* < 0.05, F_1_ = 4.181) in the main root. In addition, RKN-infection positively affected accumulation of the benzenic GSL NAS (Fig. 3E, *Mi*; *P* < 0.05, F_1_ = 5.216), whereas herbivory by *D. radicum* alone did not affect the concentration of this GSL. In terms of indole GSLs, *D. radicum* herbivory increased the accumulation of the indole GSLs I3M (Fig. 3F, *Dr*; *P* < 0.05, F_1_ = 4.539), 4OH-I3M (Fig. 3G, *P* < 0.001, F_1_ = 36.572), 4MO-I3M (Fig. 3H, *P* < 0.001, F_1_ = 45.772) and 1MO-I3M (Fig. 3I, *P* < 0.001, F_1_ = 42.362). RKN-infection positively affected the accumulation of I3M (Fig. 3F, *Mi*; *P* < 0.05, F_1_ = 4.539), 4MO-I3M (Fig. 3H  *P* < 0.05, F_1_ = 6.242) and 1MO-I3M (Fig. 3I, *P* < 0.01, F_1_ = 6.358). We did not observe any interaction effects between RKNs and *D. radicum* feeding on the accumulation of indole GSLs in the main root.

The effects of *D. radicum* herbivory and *M. incognita* infection on GSL accumulation were reflected in the expression of biosynthesis genes early after the onset of *D. radicum* herbivory. The expression of the aliphatic GSL biosynthesis gene *CYP83A1* was initially upregulated by *D. radicum* feeding (Fig. 3J, *Dr*; *P* < 0.05, F_1_ = 10.8289) but downregulated at the 48-h time point. In addition, we found an interaction effect between herbivory and RKN-infection (*Mi* × *Dr*; *P* < 0.01, F_1_ = 11.615), whereby downregulation of *CYP83A1* expression occurred earlier in the combined RKN and *D. radicum* herbivory treatment than in plants infested with *D. radicum* alone. *M. incognita* infection alone upregulated *CYP83A1* expression at the 48-h time point (*Mi*; *P* < 0.05, F_1_ = 4.798). We observed a positive effect of *D. radicum* herbivory on the expression of the indole GSL biosynthesis gene *CYP79B2* (Fig. 3K, *Dr*; *P* ≤ 0.001, F_1_ = 84.347). *M. incognita* infection did not affect *CYP79B2* expression (*Mi*; *P* = 0.201, F_1_ = 1.713), although we did observe an interaction effect between RKNs and *D. radicum*, resulting in lowered *CYP79B2* expression in these plants compared to those exposed to *D. radicum* herbivory alone at the 48-h time point (interaction effect *Mi* × *Dr*; *P* < 0.05, F_1_ = 4.394).

### Nematode infection intensifies accumulation of JA-Ile in response to *D. radicum* herbivory

Root herbivory by *D. radicum* induced an increase in the accumulation of the jasmonates JA (Fig. 4A, Supplementary Table S5; *Dr*; *P* < 0.000, F_1_ = 20.421) and JA-Ile (Fig. 4B, *Dr*; *P* < 0.01, F_1_ = 6.381) at both 24 and 48 h after the start herbivory. RKN-infection systemically increased the accumulation of JA-Ile (Fig. 4B, *Mi*; *P* < 0.05, F_1_ = 4.795), especially after 48 h (Fig. 4B, *Mi* × T; *P* < 0.05, F_1_ = 5.107). We did not observe interaction effects between *D. radicum* herbivory and RKN-infection on JA or JA-Ile accumulation. Although concentrations of SA differed between time points (Fig. 4C, T; *P* < 0.000, F1 = 15.988), neither *D. radicum* nor *M. incognita* had an effect on its accumulation. We did not observe an effect of *D. radicum* herbivory or RKN-infection on IAA concentrations (Fig. 4D). RKN-infection increased accumulation of ABA at the 48 h time point (Fig. 4E, *Mi* × T; *P* < 0.05, F_1_ = 4.257).

## Discussion

In this study, we tested whether root-feeding organisms, similar to their aboveground counterparts, can influence each other's performance via plant-mediated interactions. To do so, we assessed the performance of the cabbage root fly (*D. radicum*) on the main root of field mustard (*B. rapa*) plants, whose fine roots were infected by the root-knot nematode (*M. incognita*). *M. incognita* infection significantly increased the performance of *D. radicum*, evidenced by the higher percentage of *D. radicum* adults emerging from *M. incognita*-infected plants compared to control plants, and a higher adult body mass. We identified multiple physiological changes in the main root that potentially underlie this increase in *D. radicum* performance. We detected lower concentrations of aliphatic GSLs in the main root of RKN-infected plants compared to uninfected plants during *D. radicum* herbivory. The attenuated production of aliphatic GSLs coincided with increased levels of the defense-related phytohormone JA-Ile, and a stronger induction of indole GSLs in response to *D. radicum* in RKN-infected plants compared to noninfected plants. In addition, we observed an increase in total protein levels in the main root of RKN-infected plants but no or only minor effects on the concentrations of essential AAs and soluble sugars. Together, our results provide evidence that infection of fine roots by RKNs can facilitate the performance of an insect herbivore feeding on the main root, likely resulting from a systemic suppression of antiherbivore defenses and increased protein levels.

### Nematode infection facilitates the development of *D. radicum* larvae on *B. rapa* roots

Our results indicate that conditions for larval development were more favorable in RKN-infected compared to uninfected plants. The observed increase in the percentage of emerged flies and their higher body mass are both proxies for Darwinian fitness. Larger adult insects generally live longer (Khazaeli et al. 2005), can travel longer distances during foraging (Greenleaf et al. 2007), are less vulnerable to predators, and have higher reproductive success (Partridge and Farquhar 1983). For females, a larger body size generally corresponds with greater production of eggs (Tammaru et al. 1996; Lefranc and Bundgaard 2000), which has also been observed for the onion fly *Delia antiqua*, a congeneric of *D. radicum* (Robinson and Zurlini 1981). We observed that the developmental time from egg to adult was slightly longer for female flies emerging from RKN-infected plants compared to control plants. The timing of adult emergence can negatively affect fitness when the time lag between the emergence of females and males becomes too large, leading to impaired mating success (Tikkanen et al. 2000). In addition, longer development can reduce the number of completed generations in a season, which can affect population development of the species (Moreau et al. 2006). However, the slight difference in development time we observed between males and females on RKN-infected plants (∼2 days) is likely not critical, since the adult stage of *D. radicum* females generally lasts around 45 days (Finch and Coaker 1969; Fagerström and Wiklund 1982). To confirm whether the effects of RKN-infection indeed impact *D. radicum* fitness, follow-up studies should assess additional estimators of reproductive success, including the duration of the egg-laying period, the number of eggs present in the female and achieved fecundity over multiple generations (Moreau et al. 2006).

### Accumulation of primary metabolites is only slightly affected by nematode infection

Plant-mediated interactions may result from various underlying phenotypic changes, including differential accumulation of primary metabolites in distal tissues. For insect herbivores, larval development depends on the nutritional quality of the ingested diet, determined by the palatability of plant tissues and concentrations of essential dietary components, including AAs, proteins, and carbohydrates in the form of soluble sugars (Scriber and Slansky 1981; Ojeda-Avila et al. 2003; Fricke et al. 2008; Behmer 2009; Chapman 2012; Brunner et al. 2014; Hildebrandt et al. 2015). When present in the appropriate ratios, increased intake of these primary metabolites may result in higher larval performance (reviewed by Behmer 2009). We observed that the main roots of RKN-infected plants contained increased concentrations of proteins and 2 AAs compared to noninfected plants. In contrast, soluble sugar levels were not affected.

The increased protein levels observed in the main roots of RKN-infected plants are a possible explanation for the increased performance of *D. radicum*. Insect development greatly depends on the amount of ingested nitrogen, of which proteins are an important source (Schoonhoven et al. 2005). Plant tissues, in particular roots, are a suboptimal food source for insect herbivores because they contain relatively low concentrations of proteins (Hunter 2001; Van Dam 2009; Hildebrandt et al. 2015). An induced increase in protein levels in the food source could alleviate this limiting factor and, as such, enhance herbivore performance.

Systemically increased protein levels in roots caused by RKN-infection have been observed in other study systems and are attributed to the dialogue between RKNs and host plants during infection. This dialogue is shaped by the secretion of effector proteins by the root-knot nematode (reviewed in Goverse and Mitchum 2022) and the response of the host plant in terms of the production of resistance (R)-proteins (reviewed in Ngou et al. 2022). Indeed, infection by the soybean cyst nematode (*Heterodera glycine*) increases protein concentrations in systemic parts of the root system of soybean plants, mostly due to increased accumulation of R-proteins (Afzal et al. 2009). Such defense-related proteins are generally poorly hydrolyzable by the proteolytic enzymes of insects, complicating their breakdown into reusable AAs (Chen et al. 2005, 2007; Felton 2005). Whether the increased protein concentrations observed in this study have a similar origin and whether they benefit *D. radicum* is yet to be confirmed.

In addition to protein levels, we detected and analyzed the dynamics of 8 AAs essential for insect development (Chapman 2012). Increased amino acid levels in RKN-infected root systems have been observed in other studies, whereby the accumulation levels in systemic tissues are usually considerably lower relative to those at the nematode feeding site (Hofmann et al. 2010). We found increased concentrations of leucine and lysine in the roots of RKN-infected plants, both essential for insect development (Chang 2004). Indeed, larvae of *D. antiqua* (Diptera: Anthomyiidae) that feed on an artificial diet lacking leucine or lysine do not reach the pupal stage (Friend et al. 1957). Increased concentrations of leucine and lysine may have contributed to the increased production of proteins and enhanced herbivore performance we observed (Hildebrandt et al. 2015). However, studies on other dipteran species suggest that an unbalanced increase of a few AAs is not necessarily indicative of increased nutritional quality. In black soldier flies, supplementation of lysine to the diet actually leads to reduced larval weight and survival (Koethe et al. 2022), implying that increased concentrations of single AAs may even decrease performance. Thus, whether increases in 2 of the 10 essential AAs could have enhanced performance of *D. radicum* larvae has to be further analyzed, for example, using artificial diets (Sontowski et al. 2022).

Additionally, we observed higher concentrations of histidine and tryptophane in the main root during *D. radicum* herbivory. AAs serve as biosynthetic precursors of several defense-related secondary metabolites, including glucosinolates (reviewed by Bell 2019). High concentrations of particular AAs may indicate increased production of these chemical defenses. In *B. rapa* and other Brassicaceae, tryptophane is the biosynthetic precursor of indole GSLs that are generally induced by herbivory (Textor and Gershenzon 2009; Touw et al. 2020). Increases in tryptophane, therefore, likely result from induction of the biosynthetic pathway of indole GSLs, which indeed accumulated to higher concentrations in the *D. radicum* treatment groups.

Lastly, although root knots accumulate high concentrations of soluble sugars during the galling stage of the infection cycle (Hofmann et al. 2009; Cabello et al. 2014), we did not detect any effects of RKN-infection on sugar accumulation in the main root. This observation contrasts a study on *Arabidopsis thaliana*, where infection by the beet cyst nematode *Heterodera schachtii* induced the accumulation of multiple sugars in systemic root parts (Hofmann et al. 2010), several of which we did not detect in our analyses. These differences may be due to the differences in feeding style between cyst nematodes and root-knot nematodes (reviewed in Siddique and Grundler 2018) or to differences in methods for sugar analysis between the 2 studies.

In contrast, we found that *D. radicum* herbivory negatively affected sugar accumulation. Decreases in soluble sugar levels in response to *D. radicum* feeding, and the congeneric *D. floralis*, have been observed in several *Brassica* species (Hopkins et al. 1993, 1999), possibly because carbohydrates are remobilized from the site of herbivory toward undamaged (shoot) tissues (Robert et al. 2014), a mechanism that increases plant tolerance to herbivory.

### Nematode infection modulates defense responses to *D. radicum*

An additional explanation for the observed increase in herbivore performance may be the attenuated accumulation of defense-related secondary metabolites, such as GSLs, in the main root. Damage of *B. rapa* root tissues by *D. radicum* sets off the myrosinase-GSL defense system characteristic for the Brassicales, resulting in the hydrolysis of aliphatic GSLs and the formation of ITCs in the main root (Crespo et al. 2012). Multiple studies have observed negative effects of ITCs from aliphatic GSLs on the performance of *D. radicum* larvae (Karssemeijer et al. 2022; Sontowski et al. 2022). The decreased accumulation of aliphatic GSL we observed in the main roots of RKN-infected plants, therefore, offers an additional explanation for the facilitation of *D. radicum* larvae.

As for many other defense-related secondary metabolites, the regulatory pathways of GSL biosynthesis are activated by increased phytohormone concentrations. Therefore, we considered changes in the concentrations and mix of phytohormones in the main root due to RKN-infection as a potential mechanism behind the attenuated aliphatic GSL response we observed. Both JA and SA are known to induce changes in GSL profiles, whereby JA typically has the strongest positive effect on GSL accumulation (Mikkelsen et al. 2003; Van Dam et al. 2004; Textor and Gershenzon 2009; Guo et al. 2013; Frerigmann and Gigolashvili 2014; Augustine and Bisht 2015). The indole GSLs I3M GSL and 1MO-I3M GSL respond strongly to JA accumulation, whereas 4MO-I3M GSL is induced by SA (Liang et al. 2006; Kim and Jander 2007; Augustine and Bisht 2015). In contrast, concentrations of aliphatic GSLs are less responsive to JA treatment, which typically remain unaltered or increase by 1- to 2-fold to return to pre-induction levels at later time points (Van Dam et al. 2003). Treatment with SA may also induce increases in indole and aliphatic GSL levels, albeit much smaller (1- to 2-fold) than JA induction (Van Dam et al. 2003).

We hypothesized that to attenuate the biosynthesis of aliphatic GSLs, the induction of defense-related phytohormones in response to *D. radicum* herbivory should be suppressed in RKN-infected plants. As for most chewing insects, *D. radicum* induces the accumulation of the wounding-inducible phytohormones JA and JA-Ile, but also of SA (Karssemeijer et al. 2020, 2022). In our study, *D. radicum* also induced increases in concentrations of JA and JA-Ile in the main root but not of SA. Although we expected that RKN-infection would suppress the accumulation of these defense-related phytohormones, this is not what we observed. In fact, the main roots of RKN-infected plants contained increased concentrations of ABA and JA-Ile, which was surprising because earlier studies have shown that *M. incognita* infection may suppress JA and SA biosynthesis (Gheysen and Mitchum 2019). This discrepancy between the observations of the present and previous studies can be explained by the life stage of the nematode. Most studies addressing the effects of RKN-infection on phytohormone induction have focused on the early stages (3 to 7 days after inoculation) of nematode infection (Kyndt et al. 2012; Kammerhofer et al. 2015), whereas, in our study, the juveniles were in the later stages of their development (14 days after inoculation) and had already established their feeding site. Recent research in tomato plants has shown that the plant response to *M. incognita* infection differs greatly between life cycle stages (Mbaluto et al. 2020). During the infestation stage, *M. incognita* juveniles do not induce phytohormonal changes because they migrate intercellularly without disturbing tissues. Additionally, in the infestation and galling stages, the RKN suppresses phytohormone production by secreting effector proteins. In contrast, the life cycle transition from the galling to the reproduction stage results in the increased accumulation of several defense-related phytohormones in the aforementioned study, including SA, JA, and ABA, an effect we observed for ABA and JA-Ile. Based on the observed patterns of phytohormone induction, we rejected our hypothesis that the attenuation of aliphatic GSL concentration resulted from the suppression of defense responses to *D. radicum* due to RKN-infection.

Nevertheless, the stronger accumulation of JA-Ile in response to *D. radicum* in RKN-infected plants and the responsiveness of indole GSLs to this phytohormone may have indirectly affected aliphatic GSL concentrations in the main root via antagonisms between biosynthesis pathways of both GSL classes. In *A. thaliana*, such antagonistic relations exist between indole GSLs and aliphatic GSLs, especially butyl-GSLs such as glucoerucin, gluconapin, and progoitrin (Textor et al. 2007). Attenuation of aliphatic GSL biosynthesis thereby only occurs after strong induction of the indole GSL biosynthesis pathway, as exemplified by over-expressing genes of the indole GSL biosynthesis pathway in *A. thaliana* (Malitsky et al. 2008). Herbivory by chewing insect herbivores, including *D. radicum*, can induce such strong increases in accumulation of indole GSLs (particularly I3M GSL and 1MO-I3M GSL), leading to a decline in aliphatic GSL concentrations (Martin and Müller 2007; Textor and Gershenzon 2009; Sontowski et al. 2019; Touw et al. 2020; Karssemeijer et al. 2022). Organisms belonging to other feeding guilds may induce changes in GSL profiles differently. For example, phloem-feeders such as aphids induce the accumulation of indole GSLs (especially 4MO-I3M GSL) but not of aliphatic GSLs (Kim and Jander 2007). Belowground, cyst nematodes and root-knot nematodes such as *M. incognita*, induce systemic biosynthesis of indole GSLs in the root system (Teixeira et al. 2016; Shah et al. 2017).

In accordance with previous studies (Touw et al. 2020; Karssemeijer et al. 2022), we observed an increased accumulation of several indole GSLs in the main root (I3M, 4OH-I3M, 4MO-I3M, 1MO-I3M) during *D. radicum* herbivory. In contrast, concentrations of aliphatic or benzenic GSLs were not affected. The lack of responses in the aliphatic and benzenic GSLs that was observed in earlier studies (Karssemeijer et al. 2022) can be explained by a lower severity of herbivory due to the lower number of *D. radicum* larvae (3 vs 5 larvae) used in the present study. RKN-infection induced local increases of several GSLs in the fine roots, including the aliphatic GSL PRO, the benzenic GSL NAS, and the indole GSLs 1MO-I3M and 4OH-I3M. The positive effects on indole GSL accumulation (I3M, 4MO-I3M, 1MO-I3M) thereby extended to the main root, which is in line with earlier observations in *A. thaliana* (Teixeira et al. 2016; Shah et al. 2017).

Although neither of the individual organisms induced a decline in aliphatic GSLs, the combined treatment of *M. incognita* and *D. radicum* negatively affected the accumulation of the butyl GSL GNA and the pentyl GSL GBN. Simultaneously, the combined treatment of *M. incognita* and *D. radicum* led to a stronger accumulation of the indole GSLs I3M, 1MO-I3M, and 4MO-I3M than in either of the treatments alone. These results are in line with the stronger induction of JA-Ile that we observed in response to *D. radicum* herbivory in RKN-infected plants since this phytohormone is known to strongly induce the accumulation of indole GSLs (Textor and Gershenzon 2009). In accordance with the changes in GSL patterns, we observed an accelerated downregulation of the aliphatic GSL biosynthesis gene *CYP83A1* in response to *D. radicum*, occurring after 24 h in RKN-infected plants. In contrast, we observed no effect of RKN-infection on the induced expression of the indole GSL biosynthesis gene *CYP79B2* in response to *D. radicum* at the 24-h time point, whereas, after 48 h, expression was even lower in RKN-infected compared to noninfected plants. Although the observed expression patterns of *CYP79B2* in RKN-infected plants contradict the measured GSL-accumulation patterns, it is possible that the differential gene expression responsible for the changes in GSL concentrations occurred at an earlier time point (Schuman and Baldwin 2016). Additionally, the transcription factors that potentially mediate antagonisms between the aliphatic- and indole GSL pathways factors (reviewed Gigolashvili et al. 2009) are induced in the early phases of the plant immune response (reviewed in Spoel and Dong 2012). Follow-up studies should, therefore, include earlier sampling time points to investigate the early phases of defense induction by *D. radicum* in RKN-infected plants. Based on our results, we hypothesize that the combined effects of RKN-infection and *D. radicum* herbivory led to increased accumulation of JA-Ile and, consequently, stronger induction of indole GSLs in the main root compared to *D. radicum* herbivory alone. The induction of indole biosynthesis in turn reduced aliphatic GSL levels through antagonistic relations between the indole and aliphatic GSL biosynthetic pathways.

## Conclusions

**Figure 5.**
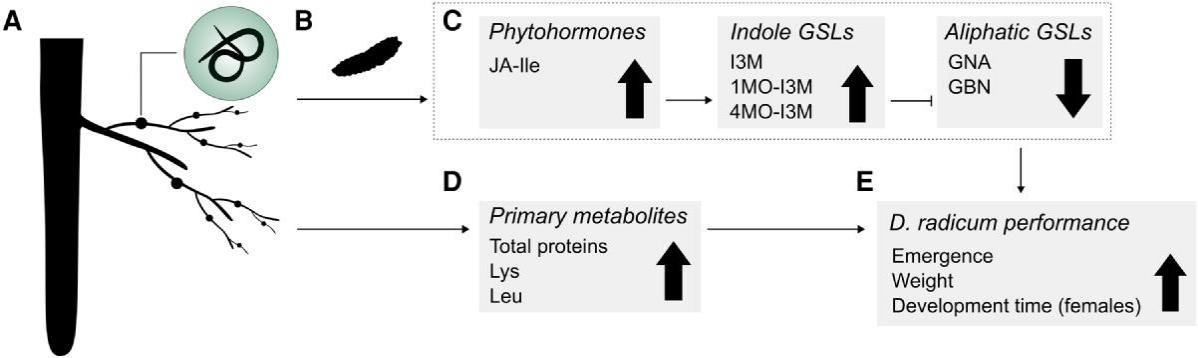
Schematic representation of the effects of *Meloidogyne incognita* infection on the induced response to *Delia radicum* herbivory in main roots of *Brassica rapa.* Schematic representation of the effects of *Meloidogyne incognita* infection on the induced defense response to herbivory by *D. radicum* larvae, which may underly the observed facilitation effect on *D. radicum*. In plants infected by *M. incognita*  **A)**, herbivory by *D. radicum* larvae **B)** leads to increased jasmonic acid isoleucine (JA-Ile) accumulation **C)** compared to noninfected plants. The higher levels of JA-Ile result in higher concentrations of indole GSLs, including indol-3-ylmethyl-GSL (I3M), 1-methoxy-indol-3-ylmethyl-GSL (1MO-I3M), and 4-methoxy-indol-3-ylmethyl-GSL (4MO-I3M). Through antagonistic interactions between the biosynthetic pathways of aliphatic and indole GSLs, the increased accumulation of indole GSLs resulted in attenuated concentrations of gluconapin (GNA) and glucobrassicanapin (GBN). Additionally, the roots of *M. incognita*-infected plants contained higher total proteins levels and higher concentrations of the essential amino acids Lysine (Lys) and Leucine (Leu) **D)**. Because the isothiocyanates that are formed during enzymatic hydrolysis of aliphatic GSLs confer resistance to chewing herbivores, decreased concentrations of these compounds may have led to the increased performance of *D. radicum* we observed in our study **E)**, together with the higher total proteins levels and concentrations of Lys and Leu.

In conclusion, this study shows that infection of the fine roots of *B. rapa* plants by the root-knot nematode *M. incognita* facilitates the development of *D. radicum* larvae feeding on the main root of the same plant. Simultaneously, RKN-infection increased total protein levels and concentrations of the essential amino lysine and leucine, and modulated the defense response of the plant to *D. radicum* herbivory in the main root (Fig. 5). We hypothesize that these induced metabolic changes underlie the observed facilitation effect on *D. radicum* performance. So far, plant-mediated interactions between plant-parasitic nematodes and insect herbivores have been assessed primarily between above- and belowground tissues (Kaplan et al. 2008; Van Dam et al. 2018; Mbaluto et al. 2021). Our study reveals that they occur within the root system as well. RKN-infection altered plant responses to *D. radicum*, as observed in the differential production of phytohormones and the expression of key enzymes of GSL biosynthetic pathways. This modulation resulted in a lowered accumulation of aliphatic GSLs, potentially caused by trade-offs between indole and aliphatic GSL biosynthesis. Possibly, this trade-off is a result of the plant fine-tuning its response to combat the most damaging effect of root damage: infection by opportunistic microbial pathogens (Touw and van Dam 2023). The production of indole GSL is biosynthetically intertwined with indole-derived phytoalexins, which confer resistance to microbial pathogens (Pedras et al. 2011). Considering that the soil is teeming with microbes, larval feeding may cause infections that are potentially more harmful to the plant than the loss of biomass to herbivory. The increased levels of indole GSLs may be a marker for this optimization of defenses. To confirm this hypothesis, follow-up studies should analyze expression patterns of MYB transcription factors that regulate GSL accumulation in the main roots at earlier time points following the start of herbivory, as well as the levels of structurally related phytoalexins. In addition, RKN-infected plants contained higher protein levels and increased concentrations of 2 essential AAs. To conclude, we hypothesize that the facilitation of *D. radicum* likely results from a combination of attenuated herbivore-induced defense responses and increased nutritional value in the main root. Considering that most, if not all, plants interact with root nematodes, these effects must be considered when studying plant defenses against insect herbivores in natural and agricultural settings.

## Materials and methods

### Insects, nematodes, and plants

The cabbage root fly (*D. radicum*) culture used in this study was started in 2016 and originated from a culture kindly provided by Dr. Anne-Marie Cortesero (University of Rennes, France). The colony was maintained on kohlrabi (*Brassica oleracea*, Gongylodes group) in a climate chamber (E-36L Reach in Plant Growth Chamber, CLF Plant Climatics GmbH, Wertingen, Germany) at 20 °C (Sontowski et al. 2019).

The root-knot nematode (*M. incognita*) colony was provided by Rijk Zwaan (De Lier, The Netherlands). It was maintained on tomato plants (*Solanum lycopersicum* cv. ‘Moneymaker’) in a greenhouse chamber at the botanical gardens of Leipzig University (Germany). Nematode juveniles (J2) were collected via a modified Baermann technique (Hooper et al. 2005). Briefly, roots from 4 plants were harvested, cut into fine pieces and distributed over a sieve (1 mm mesh size, Retsch Laboratory Equipment, Haan, Germany) placed in a tray containing tap water. After 2 days, the sieve was removed, and the water was poured over a stack of 90 *μ*m and 25 *μ*m sieves (Retsch Laboratory Equipment, Haan, Germany) to collect the J2s in a 50 mL falcon tube, which was kept at 4 °C until the start of the experiments the next day. The concentration of the stock solution was determined by quantifying the number of J2s in droplets of 10 *µ*L (*n* = 10) under a stereomicroscope (Leica DM1000 LED, Leica Microsystems, Wetzlar, Germany). Following the concentration of the stock solution, we prepared an inoculation solution with a concentration of ±400 J2 *M. incognita* juveniles per mL, to which a small volume (0.04% v/v) of Tween20 surfactant (Sigma-Aldrich/Merck, Darmstadt, Germany) was added. We based the concentration of the inoculation solution on the approximate number of J2 nematodes that may be recovered from wild *Brassica* populations (Hol et al. 2016).

The field mustard (*B. rapa*) accession used in this study was collected from a wild population in Maarssen, The Netherlands, in 2009 (De Jong et al. 2013) and propagated thereafter via open pollination (Danner et al. 2015). The seeds were germinated on fine-grained vermiculite in a climate chamber (E-36L Reach in Plant Growth Chamber, CLF Plant Climatics GmbH, Wertingen, Germany) at 25 °C (16:8 h day:night) and 60% relative humidity for 2 days. After germination, the seedlings were transferred to 2.5 L pots containing a soil:sand mixture (50:50, v:v), autoclaved twice at 121 °C. During repotting, a 4 cm long segment of plastic drinking straw was dug in close to the seedling at a 60˚ angle to facilitate noninvasive nematode inoculation. The plants were kept in a greenhouse chamber in the botanical gardens of Leipzig University (Leipzig, Germany) 27 °C (day, 16 h) and 21 °C (night, 8 h) at 50% relative humidity. When the plants had developed 2 true leaves (BBCH scale 12 (Feller et al. 1995)), we inoculated 80 plants with 1 mL of the *M. incognita* inoculation solution described above, resulting in a density of approximately 400 *M. incognita* J2s per plant. Plants belonging to non-nematode treatments were inoculated with 1 mL 1 mL water–Tween20 solution.

**Figure 6.**
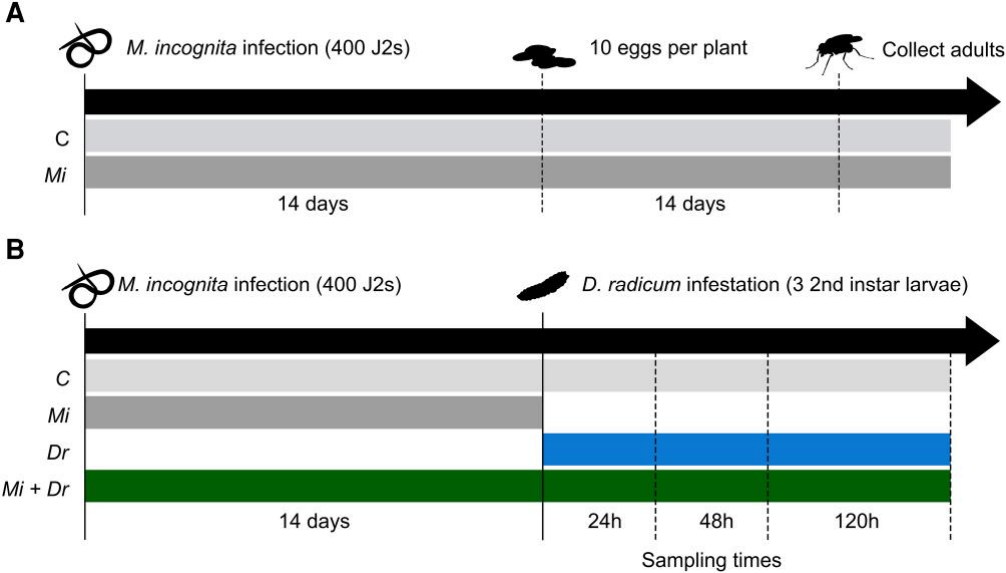
Schematic overview of experimental designs performed during the study. Schematic overview of the experimental designs of **A)** the bioassay assessing effects of infection by the root-knot nematode *Meloidogyne incognita* on the performance of the cabbage root fly *Delia radicum* and **B)** the time-series experiment that was performed to study potential effects of *M. incognita* infection on the accumulation of primary and secondary metabolites in the main root, as well as the early defense response in terms of phytohormone accumulation and expression of GSL biosynthesis genes.

All plants were placed in square plastic boxes to avoid cross-contamination between *M. incognita*-infected and uninfected plants. Of this batch, we used 10 plants during the performance assay (Fig. 6A), whereas the remaining 60 plants were used during the time-series experiment (Fig. 6B). A subset of the 10 remaining RKN-infected plants was harvested to confirm the developmental stage of *M. incognita*. The experiments started approximately 2 wk after RKN inoculation, at the time point at which the formation of root knots was confirmed.

### Root staining and determination of *M. incognita* development

To confirm the formation of root knots by *M. incognita*, we harvested the complete root systems of a separate set of 6 RKN-infected plants 14 days after inoculation. The collected roots were rinsed under running tap water to remove remaining soil particles and stained following the protocol by Ji et al. (2015). We boiled the roots in a solution of 0.8% acetic acid and 0.013% acid fuchsine for 3 min. Next, they were washed under running water and de-stained in acidified glycerol (5:100 HCl:glycerol, vol:vol) for 2 wk. The root systems were checked for root knots (see Supplementary Fig. S1B) under a stereomicroscope (Leica DM1000 LED, Leica Microsystems, Wetzlar, Germany).

### *D. radicum* performance assay

To study the effects of *M. incognita* infection on *D. radicum* performance, we infested a set of 10 RKN-infected plants and a set of 10 control plants of the same developmental age with 10 *D. radicum* eggs per plant (Fig. 6A). The eggs were part of the same oviposition batch, collected from our colony over a 2-day period. To infest the plants, we carefully removed the soil around the stem-root interface and applied the eggs to the base of the main root, which mimics the natural oviposition behavior of female flies (Zohren 1968). After replacing the sand, we placed the plants in individual foldable net cages randomly distributed over a greenhouse chamber. Starting 2 wk after adding the fly eggs, the number of emerged adults was recorded daily. Collected flies were frozen at −80 °C immediately after. The experiment stopped when no new adults had emerged for 7 days. We assessed total emergence, weight, and total development time as parameters of *D. radicum* performance. The sex of the flies was determined based on morphological characteristics described by Savage et al. (2016).

### Time-series induction experiment

We performed a time-series experiment to study the effects of *M. incognita* infection on defense responses of *B. rapa* to *D. radicum*, as well as the accumulation of primary metabolites (Fig. 6B). First, we created 3 groups of plants, each consisting of 20 RKN-infected plants and 20 uninfected control plants, corresponding to the planned harvesting time points. The plants within each group were assigned to 1 of 4 treatments (10 plants each): (i) control plants, (ii) plants infected by *M. incognita*, (iii) plants infested with *D. radicum*, and (iv) plants infected by *M. incognita* and infested with *D. radicum*. Within these groups, we created blocks of 4 plants representing the different treatments, similar in size and appearance, to be harvested simultaneously. Next, we infested the plants assigned to *D. radicum* infestation with 3 s-instar larvae by carefully removing the soil around the stem-root interface and placing the larvae directly on the main root using a fine brush. We harvested the 3 groups at 24, 48, and 120 h after the start of herbivory. During harvest, we separated the root system from the stem at the stem-root interface using a sharp scalpel. Next, the root systems were cleaned under running water and divided into 2 parts, the main root, and the lateral/fine roots, which were separately wrapped in labeled aluminum foil and flash-frozen in liquid nitrogen. We pooled the root samples of 2 plants of the same treatment and harvest date per root class into 1 biological sample. The pooled samples were ground to a fine powder under liquid nitrogen, resulting in 5 pooled samples per treatment, time point, and root class, and stored at −80 °C until further analysis.

### RNA extraction and gene expression analysis

We extracted total RNA from finely ground frozen main- and fine root material as in Touw et al. (2020), using a protocol adapted from Oñate-Sánchez and Vicente-Carbajosa (2008) as described in Supplementary Method S1. Next, we performed reverse transcription quantitative PCR (RT-qPCR) as described in Supplementary Method S1, using the gene-specific primers in Supplementary Table S7.

### Phytohormone extraction and analysis

We extracted phytohormones from homogenized, frozen root material according to Heyer et al. (2018), as described in Supplementary Method S2. Phytohormones were identified according to Mbaluto et al. (2020) based on retention time (RT) and mass-to-charge ratio (m/z) transition. The data was acquired and processed using Bruker MS data review software (Bruker MS Workstation, version 8.2.1). Phytohormone concentrations were calculated based on the peak area of the corresponding internal standard and the amount of frozen root material (ng mg^−1^ fresh weight).

### Glucosinolate extraction and analysis

Glucosinolate content was analyzed according to Grosser and van Dam (2017), as described in Supplementary Method S3. The desulfo-GSLs were identified based on retention time and UV-spectra compared to commercially available reference standards (Phytoplan, Heidelberg, Germany) and quantified using sinigrin as an external standard. Desulfo-GSL response factors and approximate retention times are shown in Supplementary Table S8.

### Protein extraction and measurement

Proteins were extracted from 100 mg of finely ground frozen main root material following an extraction method adapted from Jongsma et al. (1994), as described in Supplementary Method S4. The soluble protein content was measured in a microplate reader (Tecan Infinite 200 PRO NanoQuant, Tecan Trading AG, Germany) at 595 nm.

### Metabolome extraction, LC-MS measurements, and data processing

Extraction and analysis of the root metabolome was performed according to Weinhold et al. (2022), as described in Supplementary Method S5. The acquired LC-qToF-MS data were processed using Bruker Compass MetaboScape Mass Spectrometry Software, Version 4.0.1 (Build 594; Bruker Daltonik GmbH). The fragment spectra were matched with an in-house spectral library and public databases in a parallel search, as described in detail in Supplementary Method S6. The annotated features were classified (pathway, superclass, and class) according to the Natural Products ontology using the chemodiv package (Petrén et al. 2022) based on the descriptors (SMILES and InChIKeys) retrieved from the PubChem database. We selected putative primary metabolites (see Supplementary Table S4) based on the level of identification (according to the Metabolomics Standards Initiative (MSI; Sumner et al. 2007)), only including those that matched reference standards (level 1), or that were annotated based on spectral similarity with public/commercial spectral libraries (level 2). We focused on AAs, particularly the 10 that are considered essential for insect development (Chang 2004) and soluble sugars that are important energy sources for root fly larval development (Hopkins et al. 1993, 1999). In the case of the soluble sugars, we summed up the peak values of unique features classified as disaccharides according to Natural Products ontology as an estimate for their abundance in the analyzed extracts.

### Statistical analysis

All statistical analyses were performed in R studio (RStudio Team 2024), using version 4.3.0 of R (R Core Team 2024). Beforehand, normality of the data and homogeneity of variance were visually inspected using histograms, QQ-plots and residual plots and confirmed using Shapiro–Wilk test and Levene's test. When the data did not meet assumptions of normality, as was the case for gene expression, GSL accumulation data, we log2 transformed the data. The treatment effects on *D. radicum* adult performance were analyzed using a generalized linear model with Poisson distribution. Adult weight and development time, the accumulation of AAs, proteins, carbohydrates, phytohormones, desulfo-glucosinolate, and gene expression were analyzed using a linear model with nematode, herbivore and time point as factors. Boxplots were created using the “ggplot2” package (Wickham 2016) as part of the Tidyverse environment (Wickham et al. 2019). Raincloud plots were created using the “raincloudplots” package (Allen et al. 2019). Nonmetric multidimensional scaling (NMDS) plots were created using metaMDS function as part of the “vegan” community ecology package (Oksanen et al. 2022).

### Accession numbers

The accession numbers and sequences of the genes analyzed are displayed in Supplementary Table S7.

## Supplementary Material

kiaf109_Supplementary_Data

## Data Availability

All raw LC-MS data can be accessed at Zenodo (https://doi.org/10.5281/zenodo.10669708).

## Supplementary data

The following materials are available in the online version of this article.

**Supplementary Figure S1.** Confirmation of *M. incognita* infection in fine and main roots.

**Supplementary Figure S2.** NMDS plots visualizing dissimilarity of metabolomes in main roots.

**Supplementary Figure S3.** Estimates of nonessential amino acid concentrations in main roots.

**Supplementary Figure S4.** Estimates of glucosinolate concentrations in fine roots.

**Supplementary Table S1.** Outcomes of statistical tests of *Meloidogyne incognita* infection.

**Supplementary Table S2.** Outcomes of statistical tests of *Delia radicum* emergence.

**Supplementary Table S3.** Outcomes of statistical tests *D. radicum* performance.

**Supplementary Table S4.** Spectral information of detected primary metabolites.

**Supplementary Table S5.** Outcomes of statistical tests effects on accumulation of primary and secondary metabolites, phytohormones, and gene expression in main roots.

**Supplementary Table S6.** Outcomes of statistical tests effects on glucosinolate accumulation in fine roots.

**Supplementary Table S7.** Primer sequences used during RT-qPCR analyses.

**Supplementary Table S8.** Reference standards of desulfo-glucosinolates.

**Supplementary Method S1.** Detailed protocol for RNA extraction and qPCR analyses.

**Supplementary Method S2.** Detailed protocol for the analysis of phytohormone content.

**Supplementary Method S3.** Detailed protocol for the analysis of glucosinolate content.

**Supplementary Method S4.** Detailed protocol for the analysis of protein content.

**Supplementary Method S5.** Detailed protocol for the analysis of the root metabolome.

**Supplementary Method S6.** Processing of LC-MS data, feature annotation, and classification.

## Dive Curated Terms

The following phenotypic, genotypic, and functional terms are of significance to the work described in this paper:

CYP83A1 Gramene: AT4G13770
CYP83A1 Araport: AT4G13770

root AmiGo: PO:0009005

proteins CHEBI: CHEBI:36080

CYP79B2 Gramene: AT4G39950
CYP79B2 Araport: AT4G39950
