# Supplementary material for: Root-knot nematode infection enhances the performance of a specialist root herbivore via plant-mediated interactions
Source: Plant Physiol. 2025 Mar 20;198(4):kiaf109. doi: 10.1093/plphys/kiaf109 (PMC12395341; doi:10.1093/plphys/kiaf109)
Supplement: kiaf109_Supplementary_Data [file kiaf109_supplementary_data.pdf]

Supporting Information.

Root-knot nematode infection of *Brassica rapa* enhances the performance of a specialist root herbivore via plant-mediated interactions.

Axel J. Touw, Nhu Tran, Andreas Schedl, Jessil A. Pajar, Cong Van Doan, Henriette Uthe and Nicole M. van Dam.

Table of Contents

Supplementary Figure S1: Confirmation of *M. incognita* infection in fine- and main roots. 2

Supplementary Figure S2: NMDS plots visualizing dissimilarity of metabolomes in main roots. 3

Supplementary Figure S3: Estimates of non-essential amino acid concentrations in main roots. 4

Supplementary Figure S4: Estimates of glucosinolate concentrations in fine roots. 5

Supplementary Table S1: Outcomes of statistical tests of *Meloidogyne incognita* infection...................... 6

Supplementary Table S2: Outcomes of statistical tests of *Delia radicum* emergence. 7

Supplementary Table S3: Outcomes of statistical tests of *Delia radicum* performance............................. 7

Supplementary Table S4: Spectral information of detected primary metabolites. 8

Supplementary Table S5: Outcomes of statistical tests of the effects on accumulation of primary and secondary metabolites, phytohormones and gene expression in main roots. 9

Supplementary Table S6: Outcomes of statistical tests of the effects on glucosinolate accumulation in fine roots. 10

Supplementary Table S7: Primer sequences used during RT-qPCR analyses.. 11

Supplementary Table S8: Reference standards of desulfo-glucosinolates. 12

Supplementary Method S1: Detailed protocol for RNA extraction and qPCR analyses. 13

Supplementary Method S2: Detailed protocol for the analysis of phytohormone content. 14

Supplementary Method S3: Detailed protocol for the analysis of glucosinolate content. 15

Supplementary Method S4: Detailed protocol for the analysis of protein content. 16

Supplementary Method S5: Detailed protocol for the analysis of the root metabolome. 17

Supplementary Method S6: Processing of LC-MS data, feature annotation and classification. 18

Supplementary Figure S1: Confirmation of *M. incognita* infection in fine- and main roots.

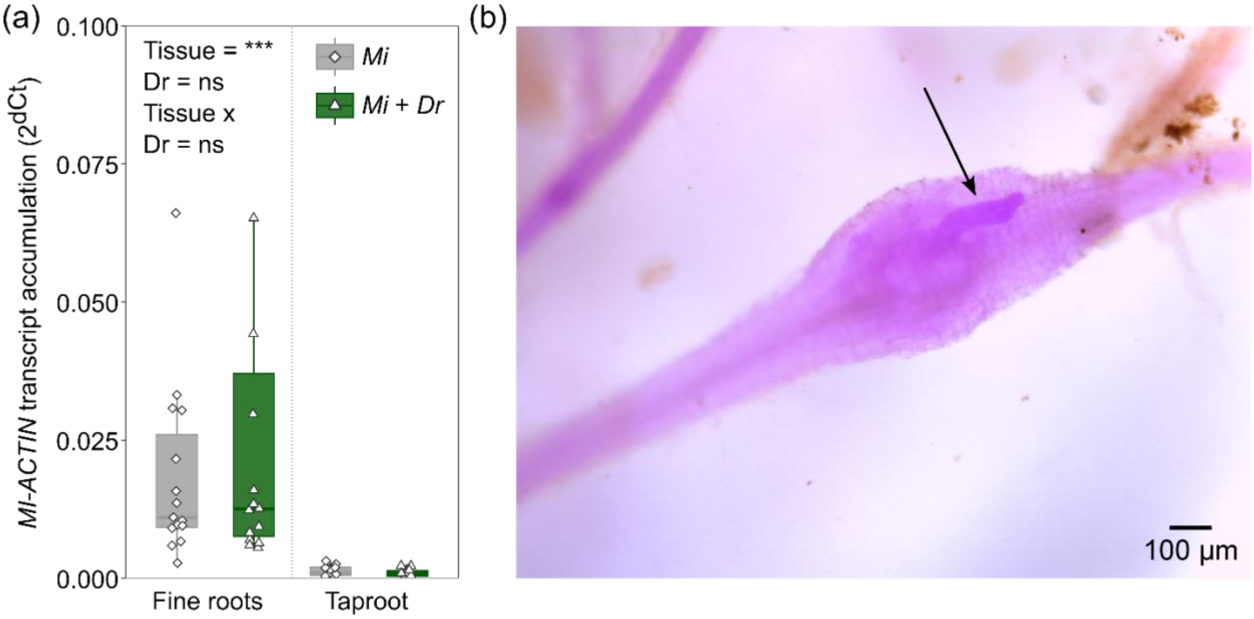

(a) Expression of *Meloidogyne incognita* actin in the fine roots and taproot of *M. incognita* (*Mi*) infected *Brassica rapa* plants infested with or in absence of *Delia radicum (Dr*). Asterisks represent statistical significance (* = p < 0.05; *** = p < 0.01, *** = p < 0.001, according to a linear model with *Mi*, *Dr* and timepoint as factors. Boxplots represent 25th to 75th percentiles and median, whiskers the 10th and 90th percentiles. Symbols represent individual replicates per treatment group and tissue (n=15). Asterisks depict statistical significance according to 2-way ANOVA: *** = p < 0.001, ns = not significant. Only statistically significant effects are depicted in the graph panels. **(b)** Photo of a fuchsine-stained root-knot containing a non-vermiform juvenile (J3) (see arrow), observed in the fine roots of *M. incognita* infected plants 14 days after inoculation. Scale bar = 100 µm.

Supplementary Figure S2: NMDS plots visualizing dissimilarity of metabolomes in main roots.

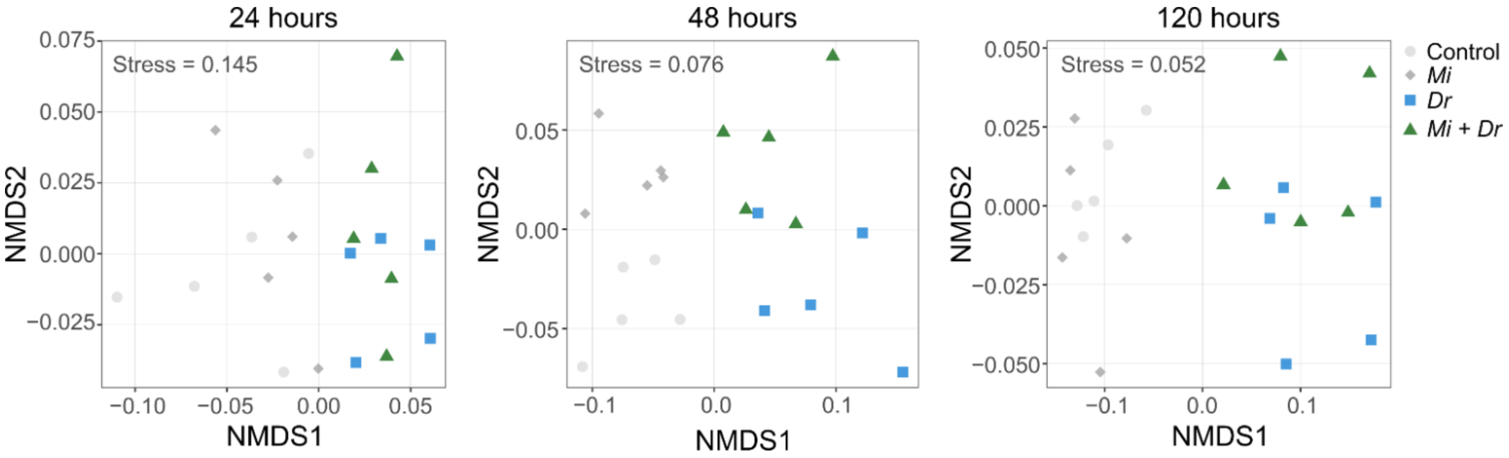

NMDS (non-metric multidimensional scaling) plots visualizing dissimilarity (according to Bray-Curtis index) of the main root metabolomes of control plants (Control; light grey dots), plants infected by *Meloidogyne incognita* (*Mi;* dark grey diamonds), plants infested by *Delia radicum* (*Dr;* blue squares), or plants infected by *M. incognita* and infested by *D. radicum* (*Mi* + *Dr*; green triangles) measured at timepoints (T) 24h, 48h and 120h after the start of *D. radicum* herbivory. Symbols represent pooled samples of two individual plants. Stress indicates goodness of fit of the model, whereby values close to 0 indicate good representation of data.

Supplementary Figure S3: Estimates of non-essential amino acid concentrations in main roots.

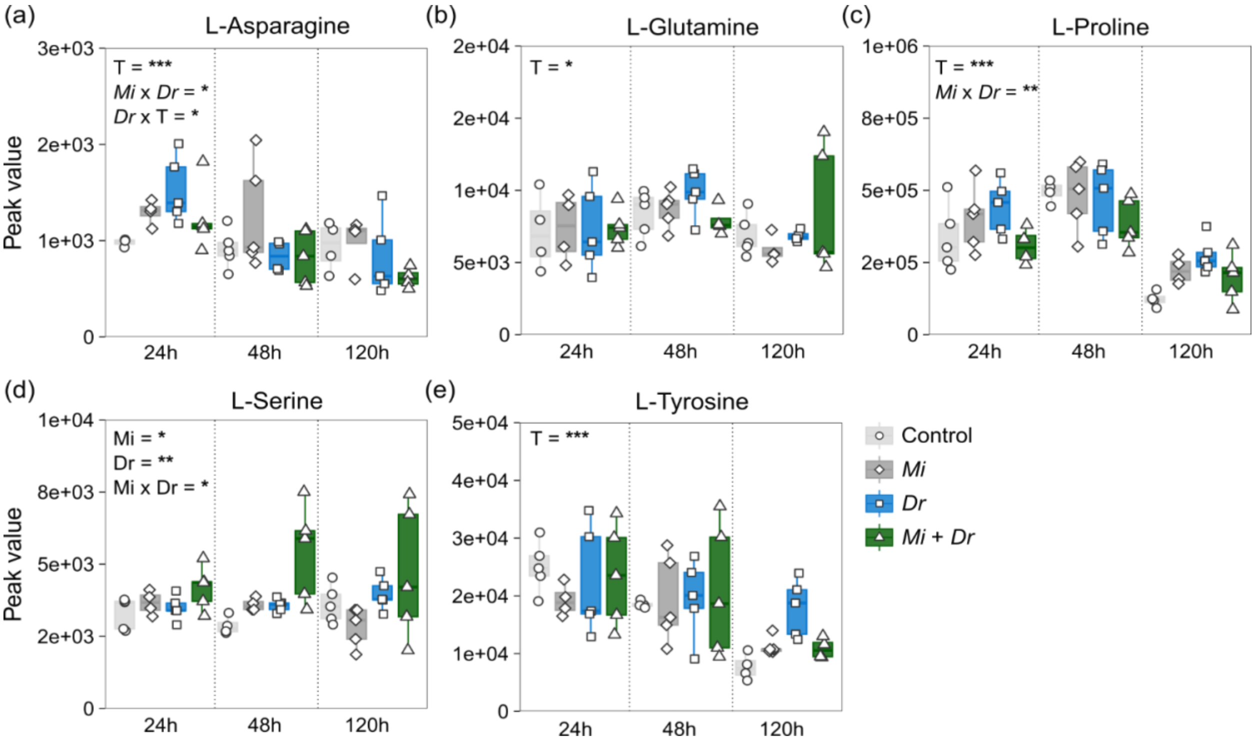

Estimates of **(a-e)** non-essential amino acids (peak value) concentrations present in the main root of control plants (Control; light grey bars), plants infected by *Meloidogyne incognita* (*Mi*; dark grey bars), plants infested by *Delia radicum* (*Dr*; blue bars), or plants infected by *M. incognita* and infested by *D. radicum* (*Mi* + *Dr*; green bars) measured at timepoints (T) 24h, 48h and 120h after the start of *D. radicum* herbivory. Boxplots represent 25th to 75th percentiles and median, whiskers the 10th and 90th percentiles. Symbols represent individual replicates per treatment group (n=5). Asterisks depict statistical significance according to 3-way ANOVA: * = p < 0.05, ** = p <0.01, *** = p < 0.0001,. Only statistically significant effects are depicted in the graph panels.

Supplementary Figure S4: Estimates of glucosinolate concentrations in fine roots.

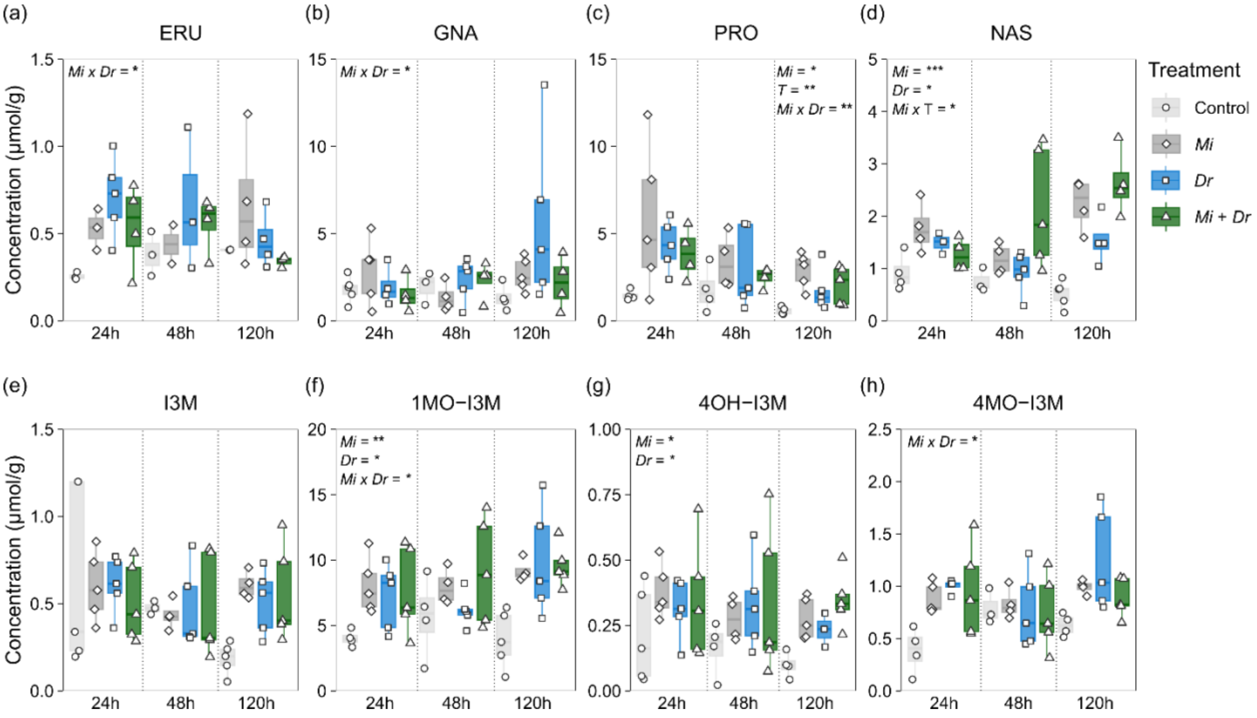

Fine root concentrations (µmol g^{-1}) of the aliphatic glucosinolates **(a)** glucoerucin (ERU), **(b)** gluconapin (GNA), **(c)** progoitrin (PRO), the benzenic GSL **(d)** gluconasturtiin (NAS), and the indole GSLs **(e)** indol-3-ylmethyl-GSL (I3M), **(f)** 1-methoxy-indol-3-ylmethyl-GSL (1MO-I3M), **(g)** 4-hydroxy-indol-3-ylmethyl-GSL (4OH-I3M) and **(h)** 4-methoxy-indol-3-ylmethyl-GSL (4MO-I3M). GSL accumulation was measured in the fine roots of control plants *Control*; light grey bars), plants infected by *Meloidogyne incognita* (*Mi;* dark grey bars), plants infested by *Delia radicum* (*Dr;* blue bars), or plants infected by *M. incognita* and infested by *D. radicum* (*Mi* + *Dr*; green bars) as measured at timepoints (T) 24h, 48h and 120h after the start of *D. radicum* herbivory. Boxplots represent 25th to 75th percentiles and median, whiskers the 10th and 90th percentiles. Symbols represent individual replicates per treatment group (n=5). Asterisks depict statistical significance according to 3-way ANOVA: * = $p < 0.05$, ** = $p < 0.01$, *** = $p < 0.001$. Only statistically significant effects are depicted in the graph panels.

Supplementary Table S1: Outcomes of statistical tests of *Meloidogyne incognita* infection.

Results of 3-way ANOVA used to test the effects of root tissue, *D. radicum* herbivory, harvesting timepoint and the interactions on these factors on *M. incognita* infection. Significant results are highlighted in bold.

	Tissue		*D. radicum*		Timepoint		Tissue x *D. radicum*		Tissue x Timepoint		*D. radicum* x Timepoint		Tissue *x* *D. radicum* x Timepoint	
MiActin	*p*	F_{Df1}	*p*	F_{Df1}	*p*	F_{Df2}	*p*	F_{Df1}	*p*	F_{Df2}	*p*	F_{Df2}	*p*	F_{Df2}
	**9.52E-05**	18.14	0.219	1.553	0.117	2.244	0.198	1.701	0.108	2.329	0.619	0.483	0.625	0.475

Supplementary Table S2: Outcomes of statistical tests of *Delia radicum* emergence.

Results of the generalized linear model (GLM) used to test the effects of *M. incognita* infection on the emergence of *D. radicum* adults. Significant results are highlighted in bold.

	M. incognita	
Adult emergence	p	χ^2
	**0.0307**	4.6667

Supplementary Table S3: Outcomes of statistical tests of *Delia radicum* performance.

Results of 3-way ANOVA used to test the effects of *M. incognita* infection, *D. radicum* adult sex and the interactions on these factors on *D. radicum* adult weight and development time. Significant results are highlighted in bold.

	M. incognita		Sex		*M. incognita* x Sex	
	p	F_{Df1}	p	F_{Df1}	p	F_{Df1}
Adult weight	**0.019**	5.8497	**4.50E-05**	19.53	0.994	0.0001
Development time	0.288	1.151	0.114	2.57	**0.012**	6.76

Supplementary Table S4: Spectral information of detected primary metabolites.

Spectral information for the disaccharides and amino acids identified in the extracted metabolome from the main root of *B. rapa.* Level of identification according to the Metabolomics Standards Initiative (MSI; Sumner et al., 2007).

Predicted class	Predicted compound	RT (min)	m/z measured	Level of identification	Spectral library
Disaccharides	Maltose	1.00	343.1229	1	Inhouse
	D-(+)-Raffinose	0.98	381.0788	1	Inhouse
	Fructose	0.97	203.0534	2	SEB
	D-(+)-Cellobiose	1.01	325.1125	2	NIST
Amino acids	L-Proline	1.00	116.0706	1	Inhouse
	L-Valine	1.00	118.0862	1	Inhouse
	L-Lysine, fragment	0.91	130.0864	1	Inhouse
	L-Leucine	1.47	132.102	1	Inhouse
	L-Histidine	0.82	156.0769	1	Inhouse
	L-Phenylalanine	2.52	166.0866	1	Inhouse
	L-Tyrosine	1.45	182.0816	1	Inhouse
	L-Glutamine	0.96	169.0582	1	Inhouse
	L-Arginine	0.82	175.1196	2	MassBank
	L-Asparagine	1.04	132.048	1	Inhouse
	L-Serine	0.99	106.0499	1	Inhouse
	L-Methionine	1.21	170.0453	2	MassBank
	L-Tryptophane	3.46	205.0972	1	Inhouse

Supplementary Table S5: Outcomes of statistical tests of the effects on accumulation of primary and secondary metabolites, phytohormones and gene expression in main roots.

Results of 3-way ANOVA used to test the effects of *M. incognita* infection, *D. radicum* herbivory, harvesting timepoint and the interactions of these factors on accumulation of individual amino acids, proteins, soluble sugars, glucosinolates, phytohormones and biosynthesis genes in main roots of *B. rapa*. Significant results are highlighted in bold.

		M. incognita		*D. radicum*		Timepoint		*M. incognita* x *D. radicum*		*M. incognita* x Timepoint		*D. radicum* x Timepoint		*M. incognita x D. radicum* x Timepoint	
		p	F_{Df1}	*p*	F_{Df1}	*p*	F_{Df2}	*p*	F_{Df1}	*p*	F_{Df2}	*p*	F_{Df2}	*p*	F_{Df2}
Amino acids	L-Valine	**0.041**	4.449	**0.000**	25.85	**0.000**	18.46	0.271	1.245	0.717	0.336	0.051	3.194	0.768	0.266
	L-Lysine	**0.046**	4.188	**0.015**	6.333	**0.033**	3.678	0.881	0.023	0.072	2.794	**0.001**	8.213	**0.010**	5.119
	L-Proline	0.374	0.806	0.852	0.035	**0.000**	35.92	**0.004**	9.508	0.633	0.462	0.132	2.124	0.490	0.725
	L-Leucine	0.380	0.786	0.986	0.000	**0.000**	25.28	**0.021**	5.768	0.673	0.400	0.416	0.896	0.726	0.322
	L-Histidine	0.374	0.807	**0.000**	63.90	**0.048**	3.233	0.762	0.093	0.248	1.438	**0.001**	7.538	0.563	0.582
	L-Phenylalanine	0.942	0.005	0.873	0.026	**0.000**	16.59	0.631	0.234	0.527	0.650	0.493	0.719	0.454	0.804
	L-Tyrosine	0.491	0.483	0.165	1.993	**0.000**	12.59	0.825	0.049	0.671	0.402	0.572	0.566	0.130	2.139
	L-Tryptophane	0.522	0.416	0.995	0.000	**0.000**	20.10	0.813	0.057	0.585	0.543	**0.005**	6.093	0.318	1.174
	L-Glutamine	0.853	0.035	0.350	0.892	**0.047**	3.289	0.948	0.004	0.706	0.351	0.713	0.341	0.229	1.525
	L-Methionine	0.910	0.013	**0.000**	53.85	**0.000**	14.53	0.702	0.148	0.242	1.462	**0.000**	9.482	0.330	1.137
	L-Arginine	0.178	1.896	0.784	0.076	**0.032**	3.817	0.339	0.943	0.409	0.919	0.474	0.764	0.958	0.043
	L-Asparagine	0.751	0.102	0.415	0.677	**0.000**	9.830	**0.024**	5.521	0.511	0.683	**0.042**	3.439	0.701	0.358
	L-Serine	**0.024**	5.475	**0.001**	12.04	0.785	0.244	**0.044**	4.303	0.132	2.115	0.328	1.143	0.645	0.443
Other primary metabolites	Proteins	**0.000**	21.85	0.201	1.684	0.451	0.811	0.643	0.218	0.723	0.327	**0.032**	3.711	0.793	0.233
	Soluble sugars	0.480	0.507	**0.000**	80.87	**0.004**	6.155	0.485	0.495	0.777	0.253	0.343	1.095	0.136	2.083
Glucosinolates	ERU	0.600	0.279	0.385	0.770	**0.000**	21.48	0.113	2.628	0.470	0.771	0.097	2.473	0.923	0.079
	GBN	0.560	0.345	0.245	1.386	0.051	3.178	**0.047**	4.181	0.398	0.939	0.075	2.747	0.337	1.115
	GNA	0.323	1.002	0.466	0.542	**0.002**	7.344	0.301	1.096	0.289	1.280	0.146	2.012	**0.011**	4.984
	PRO	0.780	0.079	0.425	0.648	**0.007**	5.564	0.148	2.166	0.881	0.128	0.391	0.961	0.690	0.374
	NAS	**0.027**	5.216	0.821	0.052	**0.001**	8.035	0.123	2.465	**0.020**	4.277	0.712	0.343	0.808	0.214
	I3M	**0.027**	5.261	0.053	3.948	0.290	1.273	0.426	0.645	0.125	2.179	**0.027**	3.899	0.789	0.238
	4OH-I3M	0.384	0.772	**0.000**	36.57	**0.012**	4.889	0.459	0.558	0.726	0.322	**0.007**	5.594	0.956	0.045
	4MO-I3M	**0.016**	6.242	**0.000**	45.77	**0.000**	16.57	0.980	0.001	0.650	0.435	**0.000**	33.27	0.289	1.279
	1MO-I3M	**0.015**	6.358	**0.000**	42.36	0.054	3.129	0.497	0.470	**0.015**	4.662	**0.007**	5.605	0.677	0.393
Biosynthesis genes	*CYP79B2*	0.201	1.713	**0.000**	84.35	0.229	1.506	**0.045**	4.394	0.487	0.496	0.968	0.002	0.054	4.032
	CYP83A1	0.520	0.424	**0.013**	6.985	**0.002**	11.97	**0.000**	23.37	**0.014**	6.928	**0.038**	4.754	**0.004**	9.994
Phytohormones	SA	0.123	2.521	0.075	3.410	**0.000**	15.988	0.517	0.431	0.814	0.056	0.326	0.998	0.126	2.487
	JA	0.908	0.014	**0.000**	20.421	0.551	0.364	0.327	0.993	0.156	2.113	0.761	0.094	0.811	0.058
	JA-Ile	**0.037**	4.795	**0.005**	9.153	**0.031**	5.119	0.176	1.923	**0.032**	5.107	0.734	0.118	0.078	3.335
	ABA	0.241	1.432	0.630	0.238	**0.000**	19.634	0.118	2.603	**0.048**	4.257	0.971	0.001	0.669	0.186
	IAA	0.225	1.535	0.073	3.445	0.068	3.576	0.056	3.947	0.059	3.840	0.579	0.315	0.582	0.310

Supplementary Table S6: Outcomes of statistical tests of the effects on glucosinolate accumulation in fine roots. Results of 3-way ANOVA used to test the effects of *M. incognita* infection, *D. radicum* herbivory, harvesting timepoint and the interactions of these factors on accumulation of individual glucosinolates in fine roots of *B. rapa*. Significant results are highlighted in bold.

		M. incognita		*D. radicum*		Timepoint		*M. incognita x D. radicum*		*M. incognita* x Timepoint		*D. radicum* x Timepoint		*M. incognita x D. radicum* x Timepoint	
		p	F_{Df1}	*p*	F_{Df1}	*p*	F_{Df2}	*p*	F_{Df1}	*p*	F_{Df2}	*p*	F_{Df2}	*p*	F_{Df2}
Glucosinolates	ERU	0.758	0.097	0.187	1.826	0.853	0.159	**0.018**	6.265	0.807	0.216	0.091	2.617	0.719	0.333
	GNA	0.475	0.521	0.167	1.985	0.162	1.907	**0.048**	4.130	0.437	0.846	0.177	1.807	0.136	2.099
	PRO	**0.015**	6.375	0.608	0.268	**0.002**	7.27	**0.004**	9.178	0.578	0.555	0.967	0.033	0.787	0.461
	NAS	**0.000**	32.587	0.011	7.232	0.053	3.174	0.408	0.702	**0.014**	4.770	0.106	2.387	0.058	3.077
	I3M	0.201	1.687	0.272	1.238	0.387	0.969	0.094	2.938	0.252	1.424	0.674	0.398	0.388	0.967
	4OH-I3M	**0.013**	6.718	**0.027**	5.247	0.514	0.676	0.128	2.405	0.717	0.336	0.818	0.202	0.852	0.161
	4MO-I3M	0.735	0.116	0.076	3.310	0.209	1.626	**0.017**	6.203	0.779	0.251	0.262	1.386	0.259	1.393
	1MO-I3M	**0.002**	11.181	**0.012**	6.858	0.286	1.289	**0.039**	4.483	0.941	0.061	0.352	1.070	0.196	1.694

Supplementary Table S7: Primer sequences used during RT-qPCR analyses.

ID	Accession	Type	Sequence 5' → 3'	Reference
ACTIN 7	Bra028615	Housekeeping gene	CCGATGGTGAGGACATTCAG	This paper
			ACAATACTCGGGAACACAGC	
GAPDH	At1G13440	Housekeeping gene	AGTTGTTGACCTCACGGTTAGAC	Tytgat et al., 2013
			TTCCTCCTTGATAGCCTTCTTG	
CYP79B2	At4G39950	CYTOCHROME P450, FAMILY 79, SUBFAMILY B, POLYPEPTIDE 2	AAGAGGTTGTGCTGCTCCG	Mathur et al., 2013
			TCCAAGTGAAACCTTGAAGAAGTC	
CYP83A1	At4G13770	CYTOCHROME P450, FAMILY 83, SUBFAMILY A, POLYPEPTIDE 1	CTCCTTATCCCTCGTGCTTG	Mathur et al., 2013
			TGTCGTAACCAGCGATCTTG	
Mi-ACTIN	MINC06773a	Housekeeping gene *M. incognita*	GATGGCTACAGCTGCTTCGT	Teillet et al, 2013
			GGACAGTGTTGGCGTAAAGG	

Supplementary Table S8: Reference standards of desulfo-glucosinolates.

Reference standards of desulfo-glucosinolates, their response factors at 229 nm and approximate retention times (RTs) on ThermoFisher/Dionex Ultimate HPLC platforms equipped with an C18 column (150 x 4.6 mm, 3 micrometer particle size) plus C18 precolumn (10 x 4.6 mm, 5 micrometer particle size). Eluents, gradient, column temperature, and flow rate as described in material and methods. RT is rounded to nearest 0.1 min (± 0.3 min depending on the column, eluent quality). Peaks, RTs and UV spectra of glucosinolates marked with * were compared with those of commercially available references (Phytoplan, Heidelberg, Germany).

Abbreviation	Common name	Side chain structure	RT (min)	229nm	Reference
	Aliphatic glucosinolates				
GNA	Gluconapin*	3-butenyl	12.85	1.11	EC, 1990
GBN	Glucobrassicanapin*	4-pentenyl	16.15	1.15	EC, 1990
ERU	Glucoerucin	4-methylthiobutyl	16.583	0.9	Brown et al., 2003
PRO	Progoitrin*	2(R)-OH-3-butenyl	5.67	1.09	Buchner, 1987; EC, 1990
	Indole glucosinolates				
I3M	Glucobrassicin*	indol-3-ylmethyl	17.423	0.29	Buchner, 1987; EC, 1990
4OH-I3M	4-hydroxyglucobrassicin*	4-hydroxyindol-3-ylmethyl	13.26	0.28	Buchner, 1987; EC, 1990
4MeOH-I3M	4-Methoxyglucobrassicin*	4-methoxyindol-3-ylmethyl	19.07	0.25	Buchner, 1987; EC, 1990
1MeOH-I3M	Neoglucobrassicin*	1-methoxyindol-3-ylmethyl	21.97	0.2	Buchner, 1987; EC, 1990
	Benzenic glucosinolates				
NAS	Gluconasturtiin*	2-phenylethyl	19.34	0.95	Buchner, 1987; EC, 1990

Supplementary Method S1: Detailed protocol for RNA extraction and qPCR analyses.

We extracted total RNA from ± 100 mg finely ground frozen main- and fine root material as in Touw et al. (2020), using a protocol adapted from Oñate-Sánchez and Vicente-Carbajosa (2008). The quality of extracted RNA was checked visually by gel-electrophoresis and by measuring absorbance ratios (260/230 and 260/280) using a NanoPhotometer® P330 (Implen, Munich, Germany). We treated the extracted RNA with DNAse I (Thermo Scientific, Waltham, MA, USA) following the manufacturer's instructions to remove any remaining genomic DNA, which we confirmed visually by gel-electrophoresis. First-strand cDNA was synthesized from 1 µg purified RNA using Revert Aid H minus reverse transcriptase (Thermo Scientific, Waltham, MA, USA) in a thermal cycler (Techne, Stone, UK) following the manufacturer's instructions at the following cycle conditions: 42°C for 60 min, 50°C for 15 min, and finally 70°C for 15 min.

We performed real-time quantitative PCR (RT-qPCR) procedures on a CFX384 Real-time qPCR system (BioRad, Munich, Germany) using gene-specific primers as described in table S1, at the following conditions: 2 min at 50°C, 5 min at 95°C, and 40 cycles of 30 s at 95°C, 30 s at 58°C, 45 s at 72°C. The genes we analyzed play a role in glucosinolate biosynthesis: *CYP83A1* (CYTOCHROME P450, FAMILY 83, SUBFAMILY A, POLYPEPTIDE 1) mediates the formation of methionine-derived aldoximes (Hemm et al., 2003) whereas *CYP79B2* (CYTOCHROME P450, FAMILY 79, SUBFAMILY B, POLYPEPTIDE 2) oxidizes the amino acid tryptophan to indole-3-acetaldoxime, a precursor in the biosynthesis of indole GSLs and other tryptophan-derived metabolites (Mikkelsen et al., 2000). We analyzed these genes for the 24h and 48h time points since previous studies have confirmed these are time points at which their expression is induced by root herbivory (Touw et al., 2020). We analyzed three technical replicates per gene for each of the five biological replicates. We calculated the relative expression of each target gene according to the comparative $2^{-\Delta\Delta Ct}$ method described in Livak and Schmittgen (2001), using the average expression of the housekeeping genes *GAPDH* and *ACTIN7* for normalization (Wang et al., 2016). Additionally, we analyzed the expression of the *M. incognita* housekeeping gene *ACTIN* (Martínez-Medina et al., 2017) in samples of the different root classes to confirm RKN-infection (Figure S1).

Supplementary Method S2: Detailed protocol for the analysis of phytohormone content.

For the extraction of phytohormones, we added 1 ml of extraction solution (100% MeOH, containing 40 ng of D_6-ABA, D_6-JA, D_6-JA-Ile, D_6-SA, D_5-IAA standards) to 250 mg (±5mg) homogenized, frozen root material, after which the samples were vortexed for 1 minute and shaken for 25 minutes to release the phytohormones from the tissue material. Next, the samples were centrifuged for 10 minutes (14000 rpm), after which the supernatant was transferred to a new reaction tube. After evaporating the extraction solution using a SpeedVac (Labconco Co-operation, Kansas, MO, USA), the dry pellets were re-suspended in 200 µl 100% methanol and analyzed by liquid chromatography (Bruker Advance UHPLC, Bremen, Germany) coupled to a mass spectrometer (Bruker Elite EvoQ Triple quadrupole, Bremen, Germany) (LC/MS EVOQ). Samples were injected in a randomized sequence. A Zorbax Eclipse XDB-C18 column (4.6 × 50 mm, 1.8 µm, 80 Å, Agilent Technologies, Santa Clara, CA, USA) was used for separation, using a mobile phase composed of solvent A (0.05 % (v:v) aqueous formic acid) and solvent B (0.05 % (v:v) formic acid in 100 % acetonitrile). The gradient was as follows: 0–0.5 min, 5 % B; 0.5–0.6 min, 5–50 % B; 0.6–2.5 min, 50–100 % B; 2.5–3.5 min, 100 % B; 3.5–3.55 min, 100–5 % B; 3.55–4.5 min, 5 % B at flow rate of 400 µL min^{-1}. Column temperature was kept constant at 42 °C. After separation, the compounds were nebulized by electron spray ionization in negative mode (ABA, JA, JA-Ile, and SA) and positive mode (IAA) respectively, at the following conditions: capillary voltage 4500 eV, cone gas 35 arbitrary units/350 °C, probe gas 60 arbitrary units/475 °C and nebulizing gas at 60 arbitrary units (Schäfer et al., 2016).

Supplementary Method S3: Detailed protocol for the analysis of glucosinolate content.

Approximately 2 g of freshly ground fine- and main root tissue was freeze-dried (Labconco Freezone 12 Plus freeze dry system), after which 50 mg was used for extraction. The sample material was incubated for 5 min in 1 mL 70% methanol at 90°C to prevent hydrolysis of GSLs by myrosinases and centrifuged. Next, the resulting supernatant was transferred to an ion-exchange column containing Sephadex G-25 (Merck, Darmstadt, Germany) as column material. After removing remaining polar compounds from the columns by washing with 70% methanol, sulfatase (*Helix pomatia* type H-1, Merck, Darmstadt, Germany) was pipetted onto the extracts to remove the sulphate group from the GSLs. The resulting desulfo-GSLs were eluted in ultrapure water and collected. The extracts were freeze-dried and re-dissolved in 1 mL ultrapure water. The desulfo-GSLs in the extracts were separated using a reversed phase high-pressure liquid chromatography (HPLC) set-up equipped with a photodiode array detector (PDA; Thermo Scientific Ultimate 3000 series, Thermo Fisher Scientific, Waltham, MA, USA) at wavelengths of 229 nm and 272 nm. A reversed-phase Acclaim™300 C18 column (4.6 × 150 mm, 3 mm, 300 Å, Acclaim 300, Thermo Fisher Scientific, Waltham, MA, USA) was used for separation with 100% H_2O (solvent A) and 99% acetonitrile in water (solvent B). The following separation conditions were used: equilibration at a gradient profile of 98% of solvent A for 4.3 min, followed by a gradient to 35% solvent B within 24.3 min and a hold until 29° min at 35% solvent B. Next, the gradient returned to the initial 98% of solvent A within 1 min and held at initial conditions for 10 min at a flow of 0.6 ml min^{-1}.

Supplementary Method S4: Detailed protocol for the analysis of protein content.

To do so, 0.3 ml protein extraction buffer (consisting of 50 g Polyvinylpolypyrrolidone (Thermo Fisher), 2g N-Phenylthiourea (Thermo Fisher), 5 g Sodium diethyldithiocarbamate trihydrate (Thermo Fisher) and 18.6 g Ethylenedinitrilotetraacetic acid disodium salt dihydrate (Carl Roth GmbH + Co. KG, Germany), dissolved in 1 L Tris-CL buffer (0.1 M, pH 7.6, Carl Roth GmbH + Co. KG)) was added to each sample, after which all samples were vortexed and incubated on ice for half an hour. After 20 minutes of centrifugation (12000g, 4°C), the supernatant was transferred to a new 2 mL tube containing 1.2 mL ice-cold saturated ammonium-sulphate solution (71 mg 100 mL^{-1}, Carl Roth GmbH + Co. KG) to precipitate the total protein content from the tissue material. After one hour, the samples were centrifuged for 10 min (12000g, 4°C), after which the supernatant was removed. The remaining pellet was re-suspended in 200 µL Tris-Cl buffer (0.1 M, pH 7.6). The resulting protein extracts were analysed following a method adapted from Bradford (1976). First, all samples were diluted 1:1 with Tris-Cl buffer (0.1 M, pH 7.6). Next, we prepared a dilution series of BSA protein in Tris-Cl (0.1 M, pH 7.6, Carl Roth GmbH + Co. KG) from 0.015 to 2 mg mL^{-1} as a calibration curve. The dilution series was pipetted *in duplo* onto a 96 well microplate, after which five microliters of diluted protein solution or dilution BSA were added 200 µL of ROTI® diluted 2:5.5 with Mili Q water (Carl Roth GmbH + Co. KG). The reaction was kept in a dark condition for 10 minutes before measurement.

Supplementary Method S5: Detailed protocol for the analysis of the root metabolome.

We extracted from 20 mg freeze-dried material (±1 mg) in 1 mL extraction solution consisting of 75% methanol and 25% acetate buffer (pH 4.8). After shaking the samples at 30 Hz for 5 minutes in a tissue homogenizer (Retch MM400, Retch GmbH, Haan, Germany) and subsequent centrifuging, the supernatant was transferred to a new 2 mL reaction tube. Afterwards, the pellet was re-extracted in 1mL extraction solution and both supernatants were combined. Next, 200 µL of the resulting extract was transferred to a glass HPLC vial and diluted 1:5 (v:v) by adding 800 µL of the extraction solution. Samples were stored at 4°C until LC-MC analysis.

Chromatographic separations of the metabolome extracts were performed at 40°C on an UltiMate™ 3000 Standard Ultra-High Performance Liquid Chromatography system (UHPLC, Thermo Scientific) equipped with an Acclaim® Rapid Separation Liquid Chromatography (RSLC) 120 column (150 mm × 2.1 mm, particle size 2.2 µm, ThermoFischer Scientific). The mobile phase consisted of solvents A (water/ formic acid 99.9/0.1 (v:v %)) and B (acetonitrile/formic acid 99.9/0.1 (v:v %)). We set the gradient to: 0-1 min, isocratic 95% A, 5%; 1-2 min, linear from 5% to 20% B; 2-8 min, linear from 20% to 25% B; 8-16 min, linear from 25% to 95% B; 16-18 min, isocratic 95% B; 18-18.01 min, linear from 95% to 5% B; 18.01-20 min, isocratic 5% B. The flowrate was kept constant at 0.4 ml min^{-1}. The injection volume was 5 µL per sample. Data were recorded from 0 min to 18 min, whereby the eluted compounds were detected from m/z 90 to 1,600 at a spectra rate of 5 Hz (line spectra only) using an ESI-UHR-qToF-MS (maXis impact, Bruker Daltonics) in positive ion mode with data-dependent collision-induced dissociation (Auto-MSMS mode). The instrument settings were as follows: nebulizer at 2.5 bar; dry gas, nitrogen, 11 L min^{-1}, dry temperature 220°C; capillary voltage at 4,500 V; end plate offset at 500 V; funnel 1 radio frequency (RF) at 200 Volts peak-to-peak (Vpp); funnel 2 RF at 220 Vpp; in-source collision-induced dissociation (CID) energy, 0.0 eV; hexapole RF, 120 Vpp; quadrupole ion energy, 4 eV; quadrupole low mass, 100 m/z; collision gas, nitrogen; collision energy, 10 eV; prepulse storage, 7 µs. Stepping: on; basic mode; collision cell RF, from 400 Vpp to 1,000 Vpp; transfer time, from 30 to 70 µs, timing; 50%/50%, collision energy for MSMS, 80%, timing 50%/50%. Data-dependent CID settings: intensity threshold 600, cycle time, 1 s, active exclusion on after 2 spectra, release after 0.5 min, smart exclusion, off, isolation and fragmentation settings, size- and charge-dependent, width 3– 15 m/z, collision energy 20– 30 eV, charge states included: 1z, 2z and 3z. In addition to the samples, we measured a mix of eight commercial standards (MM8, Böttcher et al., 2007), as well a quality control (QC) consisting of a representative mixture of all treatments after 20 sample runs. In addition, we measured injection blanks (ACN) after each batch of 10 samples.

Supplementary Method S6: Processing of LC-MS data, feature annotation and classification.

The acquired LC-qToF-MS data were processed using Bruker Compass MetaboScape Mass Spectrometry Software, Version 4.0.1 (Build 594; Bruker Daltonik GmbH). Mass recalibration, peak picking, peak alignment, region complete feature extraction, and grouping of isotopes, adduct and charge states was performed with the T-ReX algorithm in Metaboscape. We specified the following settings. Peak detection: intensity threshold of 1.500 counts, minimum peak length of 7 spectra, feature signal, intensity. Minimum peak length for recursive feature extraction, 7 spectra. Retention time range, 0– 15.6 min. Mass range, 75– 1,600 m/z. MSMS import method, average, grouped by collision energy. Ion deconvolution: EIC correlation, 0.8, primary ion, [M+H]+, seed ions, [M+Na]+, [M+K]+, [M+NH4]+, common ions, [M+H- H2O]+, and T-ReX-Positive Recalibration Auto-Detect. Feature filters: minimally present in 3 out of 98 samples, minimum for recursive feature extraction: present in 3 of 98. Next, we excluded features that, when divided by the maximum signal in the (ACN) blanks, had a maximum signal smaller than 3. This ultimately yielded 4047 features, of which the fragment spectra were first matched with an in-house spectral library (Döll, unpublished). Next, the spectra were matched against the following databases in a parallel search: GNPS (Global Natural Product Social Molecular Networking, https://gnps.ucsd.edu/), NIST17 (The NIST Mass Spectrometry Data Centre. US department of Commerce), MoNa (MassBank of North America, https://mona.fiehnlab.ucdavis.edu), Mass Bank EU (https://massbank.eu/MassBank/Index), EMBL metabolomics core facility library (http://curatr.mcf.embl.de), and Riken public databases (http://prime.psc.riken.jp/compms/msdial/main.html#MSP). We specified the following parameters: tolerances (narrow-wide): m/z 2.0– 5.0 mDa, mSigma 10– 20, MS/MS score 900– 800], without exact match of the precursor mass. These subsequent database searches resulted in a total of 1140 annotations. The annotated features were classified (pathway, superclass and class) according to the Natural Products ontology using the chemodiv package (Petrén et al., 2022) based on the descriptors (SMILES and InChIKeys) retrieved from the PubChem database. We selected the features predicted to be primary metabolites based on the level of identification (according to the Metabolomics Standards Initiative (MSI; Sumner et al., 2007)), only including those that matched to reference standards (level 1), or that were putatively annotated based on spectral similarity with public/commercial spectral libraries (level 2). We focused on amino acids, in particular the 10 that are considered essential for insect development (Chang, 2004) and soluble sugars that are important energy sources for insect development (Hopkins et al., 1993, 1999). In case of the soluble sugars, we summed up the peak values of unique features classified as disaccharides (see table S4) according to Natural Products ontology as a proxy for their abundance in the analyzed samples.
